# Simultaneous single-base resolution profiling of 5mC and 5hmC using BRIGHT-seq

**DOI:** 10.1093/nsr/nwag353

**Published:** 2026-06-10

**Authors:** Xiaochen Xue, Ziang Lu, Wei Yang, Shaoqing Han, Zhiying Wang, Yifan Jin, Xingxing Li, Xiang Zhou, Yafen Wang, Xiaocheng Weng

**Affiliations:** College of Chemistry and Molecular Sciences, State Key Laboratory of Metabolism and Regulation in Complex Organisms, Taikang Center for Life and Medical Sciences, Wuhan University, Wuhan 430072, China; College of Chemistry and Molecular Sciences, State Key Laboratory of Metabolism and Regulation in Complex Organisms, Taikang Center for Life and Medical Sciences, Wuhan University, Wuhan 430072, China; College of Chemistry and Molecular Sciences, State Key Laboratory of Metabolism and Regulation in Complex Organisms, Taikang Center for Life and Medical Sciences, Wuhan University, Wuhan 430072, China; College of Chemistry and Molecular Sciences, State Key Laboratory of Metabolism and Regulation in Complex Organisms, Taikang Center for Life and Medical Sciences, Wuhan University, Wuhan 430072, China; College of Chemistry and Molecular Sciences, State Key Laboratory of Metabolism and Regulation in Complex Organisms, Taikang Center for Life and Medical Sciences, Wuhan University, Wuhan 430072, China; College of Chemistry and Molecular Sciences, State Key Laboratory of Metabolism and Regulation in Complex Organisms, Taikang Center for Life and Medical Sciences, Wuhan University, Wuhan 430072, China; Institute of Stem Cell and Regeneration, Chinese Academy of Sciences, Beijing 100101, China; College of Chemistry and Molecular Sciences, State Key Laboratory of Metabolism and Regulation in Complex Organisms, Taikang Center for Life and Medical Sciences, Wuhan University, Wuhan 430072, China; School of Public Health, Wuhan University, Wuhan 430072, China; College of Chemistry and Molecular Sciences, State Key Laboratory of Metabolism and Regulation in Complex Organisms, Taikang Center for Life and Medical Sciences, Wuhan University, Wuhan 430072, China

**Keywords:** DNA modifications, 5-methylcytosine, 5-hydroxymethylcytosine, single-base resolution, simultaneous detection

## Abstract

Cytosine modifications in DNA, particularly 5-methylcytosine (5mC) and 5-hydroxymethylcytosine (5hmC), are key epigenetic marks involved in gene regulation and chromatin organization. Simultaneous and base-resolution detection of 5mC and 5hmC is essential for understanding their interplay in gene regulation. However, existing methods often rely on differential subtraction strategies, making direct and concurrent analysis within a single assay challenging. Here, we present the Base Replacement for Integrated Genome-wide Methylation and Hydroxymethylation Tracking Sequencing (BRIGHT-seq), a sequencing strategy that enables direct and simultaneous detection of 5mC and 5hmC at single-base resolution within the same DNA molecule. By integrating enzymatic and chemical treatments, BRIGHT-seq converts 5mC to adenine and 5hmC to thymine, enabling direct identification of both modifications without relying on differential subtraction. We demonstrate the applicability of BRIGHT-seq by mapping 5mC and 5hmC landscapes across human and mouse genomes. BRIGHT-seq provides a useful and high-resolution tool for epigenetic research, facilitating future studies on the roles of 5mC and 5hmC in gene regulation and disease.

## Teaser

BRIGHT-seq enables simultaneous single-base-resolution profiling of 5mC and 5hmC in one library through distinct C-to-A and C-to-T signatures, serving as a useful tool for studying DNA modification dynamics in development and disease.

## INTRODUCTION

DNA cytosine modifications, particularly 5-methylcytosine (5mC) and 5-hydroxymethylcytosine (5hmC), are key epigenetic marks that play essential roles in transcriptional regulation, cellular differentiation and organismal development [1–3]. 5mC, deposited by DNA methyltransferases, is typically associated with transcriptional repression and gene silencing [2]. 5hmC, generated from the oxidation of 5mC by ten-eleven translocation (TET) family enzymes, serves not only as an intermediate in active DNA demethylation but also as an independent epigenetic mark involved in transcriptional regulation [3]. Accumulating evidence indicates that 5hmC is enriched in transcriptionally active regions and contributes to chromatin remodeling and gene expression [4–6]. The dynamic interplay between 5mC and 5hmC is essential for maintaining epigenetic homeostasis and modulating various biological processes, including X-chromosome inactivation, genomic imprinting and transposable element suppression [7,8].

To investigate cytosine modifications on a genome-wide scale, various sequencing approaches have been developed. Bisulfite sequencing and enzymatic methyl-seq (EM-seq) are widely used for the identification of DNA methylation [9,10]; however, both methods are unable to distinguish between 5mC and 5hmC, as both modifications are read as cytosine during sequencing. As a result, these methods produce a composite methylation signal that lacks the resolution to distinguish between 5mC and 5hmC. To address this limitation, several methods have been developed to specifically detect 5mC or 5hmC with single-base resolution. For 5mC profiling, methods such as TET-assisted pyridine borane sequencing with β-glucosyltransferase blocking and direct methylation sequencing have been established [11–13]. Meanwhile, 5hmC-specific detection methods include improved bisulfite-based sequencing approaches [14,15], and bisulfite-free methods, such as Pvu-Seal-seq [16], APOBEC-coupled epigenetic sequencing (ACE-seq) [17], chemical-assisted C-to-T conversion of 5hmC sequencing [18] and chemical-assisted pyridine borane sequencing plus [19].

While previous methods enable precise detection of either 5mC or 5hmC, they cannot simultaneously profile both epigenetic marks within a single assay, limiting comprehensive investigation of their functional crosstalk. To overcome this limitation, several sequencing technologies have been developed for the concurrent profiling of 5mC and 5hmC, including simultaneous profiling of epigenetic cytosine modifications by sequencing (SIMPLE-seq) [20], six-letter sequencing [21], joint single-nucleus (hydroxy)methylcytosine sequencing [22] and DNA analysis by restriction enzyme for simultaneous detection of multiple epigenomic states [23]. However, these methods suffer from various limitations, including technical complexity, reliance on differential subtraction or the prerequisite for separate library preparations [24]. Furthermore, current approaches still have limitations in simultaneously distinguishing 5mC and 5hmC within the same sequencing read at single-molecule resolution, as both modifications typically undergo similar sequencing-induced mutation modes. These limitations underscore the critical need for a more intuitive and high-throughput approach to comprehensively profile 5mC and 5hmC in a single assay.

To address these challenges, we developed the Base Replacement for Integrated Genome-wide Methylation and Hydroxymethylation Tracking Sequencing (BRIGHT-seq), a streamlined approach for the direct and simultaneous detection of 5mC and 5hmC at single-base resolution within a single sequencing library. By leveraging distinct C-to-A and C-to-T conversion signatures, BRIGHT-seq enables genome-wide mapping of both modifications with single-base and single-molecule information. We validated BRIGHT-seq in both human and mouse cell lines, generating genome-wide maps of DNA methylation and hydroxymethylation landscapes. In particular, we identified distinct epigenetic patterns before and after differentiation of embryonic stem cells (ESCs). This method provides a practical strategy for profiling combined 5mC and 5hmC landscapes, establishing a practical platform for future epigenetic research.

## RESULTS

### Development of BRIGHT-seq

**Figure 1.**
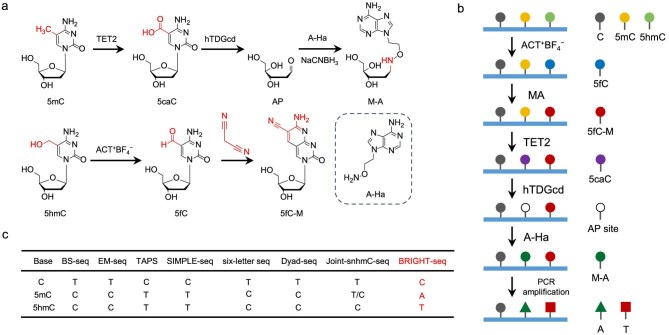
Overview of the BRIGHT-seq workflow. (a) Labeling steps for 5hmC and 5mC. (b) Schematics of BRIGHT-seq. First, 5hmC is oxidized by ACT^+^BF_4_^−^, and the newly generated 5fC is then labeled with MA to produce 5fC-M, thereby introducing a ‘C-to-T’ signal for 5hmC sites during PCR amplification. Next, 5mC is oxidized by TET2 to 5caC. Finally, hTDGcd excision and A-Ha labeling are performed to convert 5caC-derived AP sites into M-A, generating a ‘C-to-A’ signal for 5mC sites during PCR amplification. (c) Comparison of BRIGHT-seq with existing methods for 5mC and 5hmC sequencing.

We previously developed AI-seq, a base-replacement strategy for genome-wide, single-nucleotide resolution profiling of deoxyuridine (dU) [25]. In artificial incorporation modified nucleobase for sequencing (AI-seq), dU is first excised by uracil DNA glycosylase (UDG), generating an abasic (AP) site that exposes an aldehyde group in its ring-opened form [26]. This reactive site is subsequently targeted by an oxyamine-functionalized cytosine analog, N_3_-C, forming a stable adduct that enables dU detection via a T-to-C mutation during amplification. Building on this approach, we sought to extend the AI-seq strategy to detect 5mC at single-base resolution. In BRIGHT-seq, ten-eleven translocation dioxygenase 2 (TET2) and the catalytic domain of human thymine DNA glycosylase (hTDGcd) are employed to oxidize 5mC to 5-carboxylcytosine (5caC) and then excise 5caC to generate an AP site [10,27]. This site is subsequently labeled with hydroxylamine-modified adenine (A-Ha; Fig. 1a and b), resulting in a 5mC-to-A substitution during polymerase chain reaction (PCR) amplification. To detect 5hmC, we applied oxidation using 4-acetamido-2,2,6,6-tetramethyl-1-oxopiperidinium tetrafluoroborate (ACT^+^BF_4_^−^), which converts 5hmC to 5-formylcytosine (5fC) [19]. The resulting 5fC is then derivatized with malononitrile (MA) to yield a chemically stable adduct, 5fC-M [28]. During PCR amplification, 5fC-M is read as thymine (T), resulting in the conversion of 5hmC to T (Fig. 1a and b). By integrating these steps, BRIGHT-seq enables direct and simultaneous identification of 5mC and 5hmC at single-base resolution, based on distinct 5mC-to-A and 5hmC-to-T transitions (Fig. 1b). Compared to existing sequencing technologies, BRIGHT-seq leverages these two distinct base transition patterns, allowing for an intuitive and efficient readout for 5mC and 5hmC modifications without requiring differential analysis (Fig. 1c).

### Evaluation of 5mC and 5hmC labeling performance

**Figure 2.**
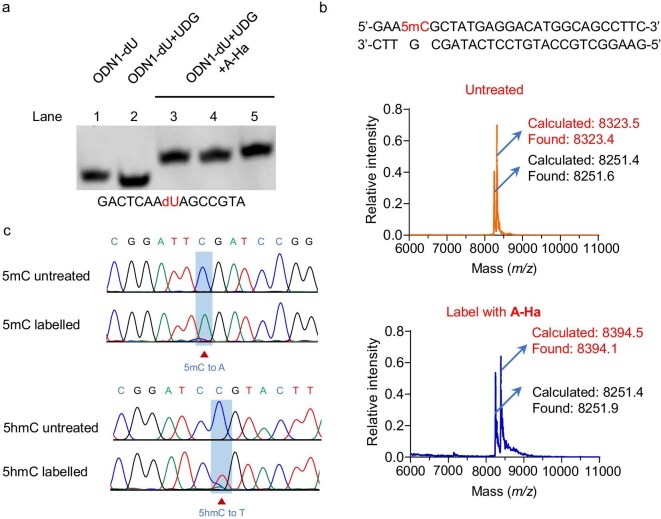
Performance of BRIGHT-seq on model DNAs. (a) Polyacrylamide gel electrophoresis (PAGE) analysis of the efficiency of A-Ha labeling for 15-nt model oligonucleotide containing an AP site. Lane 1: ODN1-dU without treatment; lane 2: ODN1-dU treated with UDG; lane 3: ODN1-AP labeled with A-Ha in 100 mM sodium acetate (NaOAc) buffer (pH 5.0); lane 4: ODN1-AP after labeling with A-Ha in 100 mM ammonium acetate (NH_4_OAc) buffer (pH 4.5); lane 5: ODN1-AP after labeling with A-Ha in 50 mM MES buffer (pH 5.0). (b) MALDI-TOF mass spectrometry characterization of 27-bp model DNA ds-ODN10-mC after A-Ha labeling of 5mC. (c) Sanger sequencing of model dsDNA containing 5mC or 5hmC, demonstrating efficient C-to-A conversion for 5mC and C-to-T conversion for 5hmC.

The efficiency of A-Ha conjugation depends on its reactivity toward AP sites. To evaluate the feasibility of A-Ha labeling, we generated AP sites by excising dU from synthetic DNA strands using UDG and optimized the labeling conditions accordingly. Gel electrophoresis and mass spectrometry analyses confirmed the formation of A-Ha–AP adducts (M-A) on single and multiple AP sites within the same DNA strand across various DNA sequences under mildly acidic conditions (Fig. 2a and Figs S1–S3 and S4a and b). Furthermore, to examine how efficiently the M-A adduct could be efficiently recognized by DNA polymerase, we performed A-Ha labeling and amplification using a 104-bp double-stranded DNA (dsDNA) model containing a single AP site flanked by randomized bases (NN-AP-NN, N = A, T, C or G; see the section ‘Materials and Methods’ and Table S1 in the Supplementary Information). We found that most AP-site contexts (165 out of 256) showed mutation rates >80%, and more than half (137 out of 256) exhibited mutation rates >85% (Fig. S5). However, ∼35% of the motifs showed conversion rates below 80%, and some motifs had efficiencies as low as 50% or even lower. These results indicate that the efficiency of conversion varies across sequence contexts, which may lead to an underestimation of 5mC modification levels at these specific motifs, potentially affecting the accuracy of 5mC detection.

We next evaluated the oxidation activity of TET2 on both a 5mC-containing model duplex DNA (ds-ODN10-mC, annealed by ss-ODN10-mC-F and ss-ODN10-R; Table S1) and genomic DNA (gDNA) from human embryonic stem cells (hESCs; H9 line). High-performance liquid chromatography–tandem mass spectrometry (HPLC–MS/MS) analysis showed that >98% of total 5mC/5hmC in H9 gDNA was converted to 5caC after TET2 oxidation (Fig. S6). Furthermore, we verified that hTDGcd possesses glycosylase activity toward 5caC in dsDNA (ds-ODN9-caC, annealed by ss-ODN9-caC-F and ss-ODN9-R; Table S1), while showing no detectable endonuclease activity at the resulting AP site (Fig. S7). We also observed that the 5mC labeling process caused moderate DNA degradation under our experimental conditions (Fig. S8), possibly due to the instability of intermediate AP sites.

Furthermore, we evaluated the whole 5mC processing workflow using model dsDNA substrates. Conversion of 5mC to M-A was confirmed by matrix-assisted laser desorption/ionization time-of-flight (MALDI-TOF) mass spectrometry (Fig. 2b), high-performance liquid chromatography–mass spectrometry (Fig. S4c and d) and gel electrophoresis (Fig. S7c). Moreover, when an 80-bp dsDNA model containing a single 5mCpG (ds-ODN12-mC, annealed by ss-ODN12-mC-F and ss-ODN12-R; Table S1) was subjected to the 5mC labeling procedure, a clear C-to-A transition (∼90%) was observed during PCR amplification, as validated by both Sanger sequencing and restriction enzyme digestion (Fig. 2c and Fig. S9a). These results supported the detection of 5mC through C-to-A conversion.

To validate the feasibility of 5hmC conversion, we evaluated the performance of our chemical strategy on a 100-bp dsDNA substrate containing a single 5hmCpG (ds-ODN11-hmC, annealed by ss-ODN11-hmC-F and ss-ODN11-R; Table S1), using ACT^+^BF_4_^−^ oxidation followed by derivatization with MA. Sanger sequencing and restriction enzyme digestion confirmed 5hmC-to-T conversion following PCR amplification (Fig. 2c and Fig. S9b), demonstrating successful oxidation of 5hmC to 5fC and the subsequent formation of the 5fC-M product, consistent with previous reports [19,28]. These results support the feasibility of our chemical labeling approach. Together, our findings show that site-specific conversions of 5mC and 5hmC were achieved in model substrates, enabling the simultaneous profiling of these two epigenetic marks at single-base resolution.

### Performance evaluation of BRIGHT-seq for simultaneous 5mC and 5hmC detection

**Figure 3.**
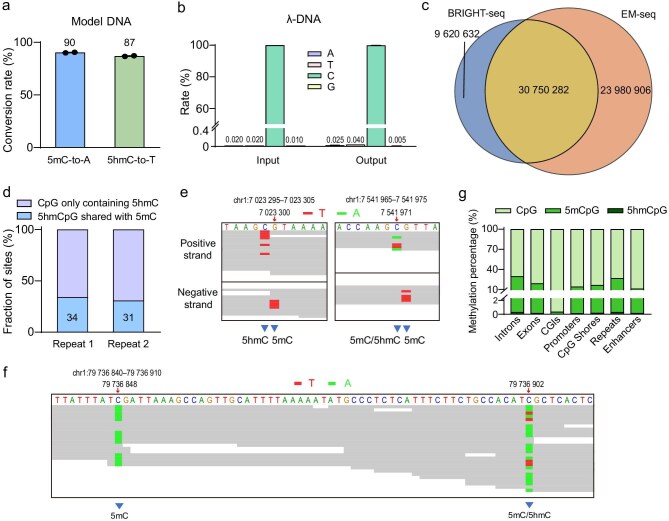
BRIGHT-seq performance on spike-in DNA and HEK293T gDNA at single-base resolution. (a) Conversion efficiencies of 5mC-to-A and 5hmC-to-T in the 116-bp spike-in dsDNA (*n* = 2). (b) Evaluation of BRIGHT-seq false-positive rates using spike-in *λ*-DNA (*n* = 2). (c) Venn diagram showing the overlap and method-specific modified sites detected by BRIGHT-seq and EM-seq after merging loci detected across replicates within each method. (d) Proportion of 5hmC sites that co-localize with 5mC at the same CpG positions in HEK293T gDNA (*n* = 2). (e and f) Integrative Genomics Viewer (IGV) showing 5mC and 5hmC at symmetric sites (e), and within the same read and at the same site (f) in the HEK293T genome. (g) Distribution of unmodified CpG, 5mCpG and 5hmCpG across different genomic features in the HEK293T genome.

To assess the performance of BRIGHT-seq for simultaneous detection of 5mC and 5hmC, we used a 116-bp synthetic dsDNA spike-in containing one 5mCpG site and one 5hmCpG site at different loci on the same strand (ds-ODN13-mChmC, annealed by ss-ODN13-F-mChmC and ss-ODN13-R-C; Table S1). Following the labeling workflow and next-generation sequencing, the conversion efficiencies were ∼90% for 5mC-to-A and ∼87% for 5hmC-to-T (Fig. 3a and Fig. S9c), indicating that the two orthogonal base-transition modes can be distinguished within the same library. We also observed minor cross-calling at the fully modified spike-in sites, with 2.69% of reads showing a C-to-T signal at the 5mC site and 6.69% showing a C-to-A signal at the 5hmC site (Fig. S9c). To minimize the impact of such events in genome-wide analyses, high-confidence 5mC or 5hmC sites were called using thresholds of mutation rate >10%, ≥ 2 mutation-supporting reads and ≥3 total reads. Importantly, analysis of a negative control (λ-DNA) revealed a negligible conversion rate for unmodified cytosines (0.025%; Fig. 3b).

We further evaluated BRIGHT-seq using synthetic dsDNA mixtures in which modified and unmodified molecules were combined to yield 10% 5mC or 10% 5hmC at the target CpG site (ds-ODN13-mC/C, annealed by ss-ODN13-F-mC or ss-ODN13-F-C with ss-ODN13-R-C, and ds-ODN11-hmC/C, annealed by ss-ODN11-hmC-F or ss-ODN11-C-F with ss-ODN11-R; Table S1). The corresponding mutation signals were observed for both 5mC and 5hmC (Fig. S10a and b). However, detectable false-positive signals were also observed. For example, at the 10% input level, the false-positive 5hmC calling rate in the 5mC/C mixture was 0.92%, and the false-positive 5mC calling rate in the 5hmC/C mixture was 0.51%. In addition, the detected C-to-A signal in the 10% 5mC/C mixture was 12.59%, higher than the theoretical input level, suggesting that BRIGHT-seq can approximately detect low-level modifications under our experimental conditions, while exact absolute quantification at this abundance remains limited.

Furthermore, by mixing synthetic dsDNAs containing 5mC and 5hmC at the same cytosine position at defined abundance ratios (5mC:5hmC = 1:3, 1:1 and 2:1; ds-ODN13-mC/hmC, annealed by ss-ODN13-F-mC or ss-ODN13-F-hmC with ss-ODN13-R-C; Table S1), we found that these mixed populations could be distinguished by BRIGHT-seq (Fig. S10c). Nevertheless, the detected C-to-A/C-to-T ratios did not exactly match the theoretical input ratios, suggesting that BRIGHT-seq can distinguish mixed 5mC/5hmC populations, but precise quantification of their relative proportions remains a limitation of the current workflow.

We next applied BRIGHT-seq to perform genome-wide profiling of 5mC and 5hmC in human HEK293T cells. Because EM-seq reports cytosine modification signals without distinguishing 5mC from 5hmC, we compared EM-seq sites with the union set of BRIGHT-seq-identified modified loci (i.e. sites called as 5mC by the C-to-A channel or as 5hmC by the C-to-T channel). After merging the modified loci detected across replicates within each method, BRIGHT-seq detected 40 370 914 modified loci in HEK293T gDNA, among which 30 750 282 overlapped with EM-seq and 9620 632 were uniquely detected by BRIGHT-seq. EM-seq detected 54 731 188 modified loci, including 23 980 906 loci uniquely detected by EM-seq (Fig. 3c). Overall, 76% of BRIGHT-seq-detected modified loci overlapped with EM-seq, suggesting considerable overlap between the two methods. The smaller number of loci detected by BRIGHT-seq may be partly attributable to the lower current labeling/conversion efficiency of the BRIGHT-seq workflow, which could lead to missed detection of some low-modification-level methylated sites. BRIGHT-seq revealed that over 30% of the detected 5hmC sites co-occurred with 5mC at the same CpG positions, supporting the notion that 5hmC serves as an intermediate in active DNA demethylation (Fig. 3d). BRIGHT-seq enabled simultaneous detection of both 5mC and 5hmC within the same DNA molecule, capturing coexisting modifications either at the same locus or on complementary DNA strands (Fig. 3e and f). These results demonstrate that BRIGHT-seq can provide single-molecule information for both 5mC and 5hmC and distinguish coexisting modification signals on the same DNA molecule. We further analyzed 5mC and 5hmC distribution patterns across genomic features. CpG islands (CGIs) consistently exhibited low levels of CpG modifications (Fig. 3g), in line with the well-established notion that CGIs are typically hypomethylated [29].

### BRIGHT-seq reveals genomic co-occurrence patterns of 5mC and 5hmC across diverse cell types

Next, we applied our sequencing technology to multiple cell lines, including HEK293T, HeLa, K562 and ESCs. Under the specific experimental conditions applied to mouse embryonic stem cell (mESC) gDNA, BRIGHT-seq achieved a unique mapping rate of ∼82% (Table S2). Notably, BRIGHT-seq currently used higher DNA input and more PCR cycles than several compared methods, which should be considered when interpreting mapping and duplication metrics across technologies. The average 5mC level measured by BRIGHT-seq at CpG sites is slightly lower than that reported by SIMPLE-seq [20], possibly owing to incomplete conversion of 5mCpG in certain sequence contexts (Fig. S5). For 5hmC, the average 5hmC level detected by BRIGHT-seq at CpG sites was comparable to the results measured by ACE-seq (Fig. S12). We next compared BRIGHT-seq with existing methods at larger genomic scales. BRIGHT-seq 5mC raw signals showed a moderate correlation with signals derived from EM-seq–ACE-seq subtraction at the 10-kb bin level (Pearson’s *r* = 0.69; Fig. S13a). In parallel, BRIGHT-seq 5hmC raw signals showed moderate correlations with ACE-seq (Pearson’s *r* = 0.41) and SIMPLE-seq (Pearson’s *r* = 0.47), comparable to that observed between published SIMPLE-seq [20] and ACE-seq 5hmC signals in mESCs at the 100-kb bin level (Pearson’s *r* = 0.30; Fig. S13b). We then performed single-CpG-level comparisons across shared CpG sites. At this resolution, the correlations were substantially lower. BRIGHT-seq 5mCpG raw signals showed a weak correlation with 5mCpG signals estimated by EM-seq–ACE-seq subtraction (Pearson’s *r* = 0.31; Fig. S14a). For 5hmCpG, BRIGHT-seq raw signals showed weak correlations with ACE-seq (Pearson’s *r* = 0.07) and SIMPLE-seq (Pearson’s *r* = 0.29) (Fig. S14b). As a reference comparison, the single-CpG correlation between two replicate 5mCpG datasets estimated by EM-seq–ACE-seq subtraction was Pearson’s *r* = 0.49, and the correlation between two ACE-seq replicates was Pearson’s *r* = 0.18 for 5hmCpG. These results indicate that the quantitative concordance of BRIGHT-seq with existing methods is more limited at individual CpG sites than at larger genomic scales. Differences in per-site read coverage across libraries may be one possible factor influencing these single-CpG-level comparisons.

**Figure 4.**
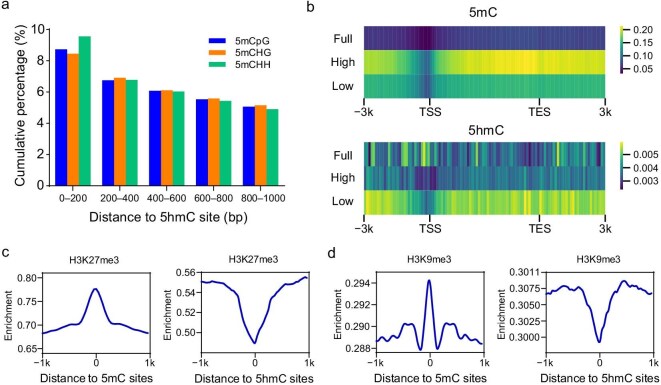
BRIGHT-seq characterization of 5mC and 5hmC distribution in K562 cells. (a) Cumulative distribution of distances from different types of 5mC sites (5mCpG, 5mCHG and 5mCHH) to the nearest 5hmC sites, with distances grouped into 200-bp bins. (b) Distribution patterns of distinct states of 5mC and 5hmC levels around TSSs and transcription end sites (TESs) (low, 10%–50% modification level; high, 50%–90% modification level; full, >90% modification level). (c and d) The relationship between modified cytosine sites and distribution patterns of histone modifications. Line plots showed the enrichment of (c) H3K27me3 from ENCODE ChIP-seq data (ENCFF470HOG) and (d) H3K9me3 from ENCODE ChIP-seq data (ENCFF559MMQ) around 5mC or 5hmC sites [30].

We observed that 5hmC sites frequently occurred in close genomic proximity to 5mC sites (Fig. 4a and Fig. S15). This pattern reinforces our earlier observation that 5hmC and 5mC often overlap at the same CpG positions (Fig. 3d), indicating a coordinated relationship between the two modifications during epigenetic regulation. Furthermore, BRIGHT-seq was able to detect clustered 5mC sites (Fig. S16) [29].

We next examined the distribution of 5mC and 5hmC within gene bodies in all analyzed cell types. Based on modification levels, cytosines were categorized into three groups: low (10%–50%), high (50%–90%) and full (>90%) modification. Across all groups, modified cytosines were rarely detected near transcription start sites (TSSs), and fully methylated sites were almost absent in these regions, consistent with the established role of 5mC in repressing transcription initiation [1]. Notably, 5hmC was consistently detected at low modification levels across all cell types (Fig. 4b and Fig. S17), likely reflecting its generally lower abundance relative to 5mC.

To further explore the relationship between 5mC, 5hmC and other regulatory elements, we integrated BRIGHT-seq data with histone modification profiles from the ENCODE database [30]. In K562 cells, histone marks H3K27me3 and H3K9me3 were enriched in regions with higher densities of 5mC. In contrast, both marks showed an inverse spatial association with 5hmC (Fig. 4c and d). These observations suggest that H3K27me3 and H3K9me3 may cooperate with 5mC, while displaying an antagonistic relationship with 5hmC. These findings provide insight into the interplay between histone modifications and cytosine methylation states.

### Cytosine modification dynamics during hESC differentiation

**Figure 5.**
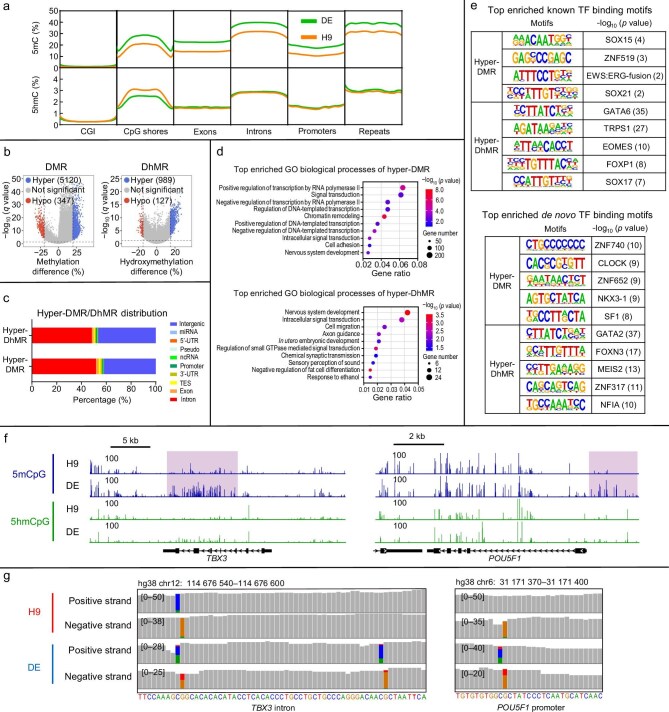
BRIGHT-seq analysis of hESC genomes before and after differentiation from H9 to DE cells. (a) Distribution of average 5mC and 5hmC levels at CpG sites across various genomic features in H9 (orange) and DE (green) cells. (b) Left: Volcano plot of DMRs. Blue dots represent hyper-DMRs (methylated CpG/CpG difference >20%; *q* ≤ 0.05), and red dots represent differentially hypomethylated regions (hypo-DMRs; methylated CpG/CpG difference <−20%; *q* ≤ 0.05). Right: Volcano plot showing differentiation-associated DhMRs. Blue dots represent hyper-DhMRs (hydroxymethylated CpG/CpG difference >15%; *q* ≤ 0.05), and red dots represent differentially hypohydroxymethylated regions (hypo-DhMRs; hydroxymethylated CpG/CpG difference <−15%; *q* ≤ 0.05). (c) Bar plots showing the distribution of hyper-DhMRs (top) and hyper-DMRs (bottom) across different genic elements. (d) Gene ontology enrichment analysis of biological processes associated with hyper-DMRs (top) and hyper-DhMRs (bottom). (e) Top enriched known and *de novo* TF motifs identified within overlapping hyper-DMRs and hyper-DhMRs. (f) IGV tracks displaying CpG modification levels at the loci of pluripotency marker genes *TBX3* and *POU5F1*. Pink boxes indicate regions with distinct methylation states before and after differentiation. (g) Representative single-base-resolution views of 5mC and 5hmC sites in genic elements of pluripotency marker genes *TBX3* and *POU5F1*.

To explore cytosine modification dynamics during early human development, we applied BRIGHT-seq to profile genome-wide 5mC and 5hmC patterns in H9 cells and their differentiated counterpart, definitive endoderm (DE) cells. The average 5mCpG levels were higher in DE cells than in H9 cells, while the average 5hmCpG levels showed no significant variation between the two cell types across most analyzed genomic features (Fig. 5a). Specifically, H9 gDNA exhibited increased hydroxymethylation levels only in CpG shores.

Differential modification analysis between H9 and DE cells revealed 5467 differentially methylated regions (DMRs) and 1116 differentially hydroxymethylated regions (DhMRs). The majority of DMRs (5120, 93.7%) and DhMRs (989, 88.6%) showed hypermodification in DE cells (Fig. 5b). These regions were termed as hypermethylated DMRs (hyper-DMRs) and hyperhydroxymethylated regions (hyper-DhMRs), which were predominantly located in intronic and intergenic regions (Fig. 5c), suggesting regulatory functions beyond promoter methylation. Gene ontology analysis revealed enrichment of hyper-DMRs in transcriptional regulation processes, while hyper-DhMRs were associated with nervous system development (Fig. 5d).

To further elucidate the regulatory roles of cytosine modifications during hESC differentiation, we investigated transcription factor (TF) motif enrichment within identified DMRs and DhMRs. Known motifs of pluripotency-associated TFs, including SOX15 and SOX21, were significantly enriched in hyper-DMRs, suggesting that DNA methylation may contribute to the repression of pluripotency networks. In contrast, hyper-DhMRs were enriched for motifs of DE-specific TFs such as GATA6, EOMES and SOX17, suggesting that 5hmC may be associated with lineage-specific transcription programs (Fig. 5e).

*De novo* motif analysis further identified NKX3-1 as a potential regulator associated with hyper-DMRs, while GATA2, MEIS2 and NFIA were enriched in hyper-DhMRs, indicating distinct epigenetic regulators involved in pluripotency exit and DE lineage commitment (Fig. 5e). Additionally, BRIGHT-seq enabled base-resolution mapping of hypermethylation in key regulatory genes, including *TBX3* gene body and *POU5F1* promoter (Fig. 5f and g). These results suggest that 5mC and 5hmC may be associated with distinct regulatory programs during DE differentiation. These findings suggest coordinated and potentially distinct roles of 5mC and 5hmC in regulating the epigenetic transitions during early endodermal differentiation.

### Cytosine modification dynamics during mESC differentiation

**Figure 6.**
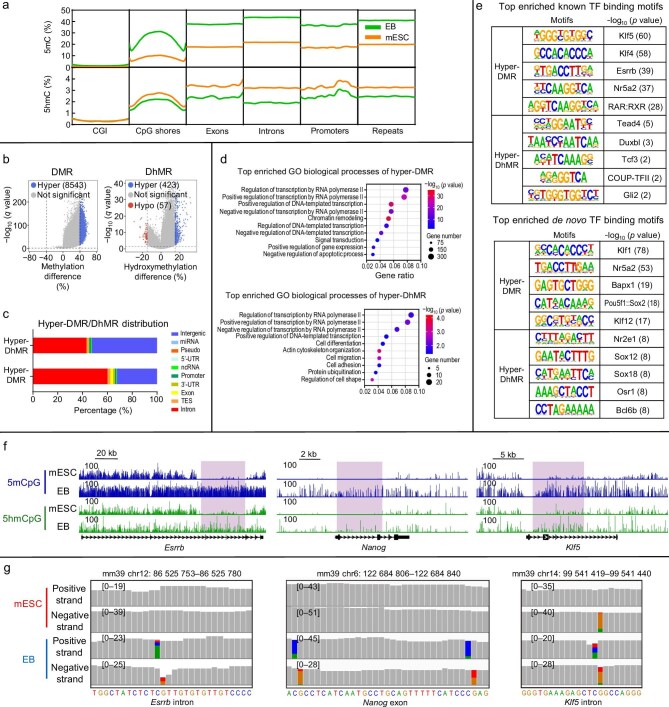
BRIGHT-seq profiling of cytosine modifications in mESC and EB. (a) Distribution of average 5mC and 5hmC levels at CpG sites across various genomic features in mESCs (orange) and EBs (green). (b) Left: Volcano plot displaying differentiation-associated DMRs in mESCs. Blue dots represent hyper-DMRs (methylated CpG/CpG difference >40%; *q* ≤ 0.05). Right: Volcano plot showing differentiation-associated DhMRs in mESC. Blue dots represent hyper-DhMRs (hydroxymethylated CpG/CpG difference >15%; *q* ≤ 0.05), and red dots represent hypo-DhMRs (hydroxymethylated CpG/CpG difference <−15%; *q* ≤ 0.05). (c) Bar plot indicating the distribution of hyper-DhMRs (top) and hyper-DMRs (bottom) in various genic elements during mESC differentiation. (d) Gene ontology enrichment analysis of biological processes associated with hyper-DMRs (top) and hyper-DhMRs (bottom). (e) Top enriched known and *de novo* TF motifs identified within overlapping hyper-DMRs and hyper-DhMRs. (f) IGV tracks displaying CpG modification levels at the loci of pluripotency marker genes *Esrrb, Nanog* and *Klf5*. Pink boxes indicate regions with distinct (hydroxy)methylation states before and after differentiation. (g) Representative single-base-resolution views of 5mC and 5hmC modifications across gene bodies of *Esrrb, Nanog* and *Klf5*.

We next examined the dynamics of DNA methylation and hydroxymethylation during *in vitro* differentiation from mESC to embryoid body (EB). The average 5mCpG levels were higher in EBs than in mESCs across a range of genomic features, except for CGIs (Fig. 6a), consistent with previous studies reporting global hypermethylation during the transition from 2i-cultured mESCs to EBs [31]. Conversely, the average 5hmC levels at CpG sites decreased in EBs (Fig. 6a), suggesting distinct dynamics of 5mC and 5hmC during mESC differentiation [2,3].

Through genome-wide differential (hydroxy)methylation analysis, we next identified 8544 DMRs and 480 DhMRs associated with differentiation. Nearly all of the identified DMRs (8543) exhibited significant hypermethylation (Fig. 6b), while the majority of DhMRs (423, 88.1%) showed hyperhydroxymethylation. Most of the hyper-DMRs and hyper-DhMRs were located within intronic and intergenic regions (Fig. 6c), a distribution pattern that closely mirrored the results observed during hESC differentiation. Gene ontology analysis showed that hyper-DMRs and hyper-DhMRs were mainly enriched in transcriptional regulation pathways, with hyper-DhMRs specifically associated with cell differentiation processes (Fig. 6d).

Motif enrichment analysis revealed that hyper-DMRs were significantly enriched for binding motifs of pluripotency-associated TFs, including Klf5, Klf4, Esrrb and Nr5a2. Hyper-DhMRs were associated with TFs that suppress pluripotency and promote differentiation, such as Tead4, Duxbl, Tcf3 and COUP-TFII. *De novo* motif analysis identified additional motifs of pluripotency-associated TFs, such as Nr5a2 and Pou5f1::Sox2, enriched in hyper-DMRs, and motifs of developmental regulators, including Nr2e1, Sox18 and Bcl6b in hyper-DhMRs (Fig. 6e). Leveraging BRIGHT-seq’s single-base resolution, we observed pronounced increases in both 5mCpG and 5hmCpG within gene bodies of *Esrrb, Nanog* and *Klf5* (Fig. 6f and g), key regulators of pluripotency. These observations demonstrate the application of BRIGHT-seq in capturing locus-specific and coordinated remodeling of cytosine modifications during EB differentiation at single-base resolution.

## DISCUSSION

In this study, we developed BRIGHT-seq, a single-base-resolution sequencing method that enables direct and simultaneous detection of 5mC and 5hmC across the genome. BRIGHT-seq utilizes mild enzymatic and chemical treatments to convert 5mC to the adenine analog A-Ha and 5hmC to 5fC-M. These modifications are subsequently read as C-to-A and C-to-T transitions during sequencing, respectively, thereby allowing identification of both epigenetic marks from a single library. Under our experimental conditions, BRIGHT-seq enabled genome-wide profiling of 5mC and 5hmC while largely preserving unmodified cytosines. In addition, this strategy can support integrated epigenomic analyses.

Compared with existing methods for joint profiling of 5mC and 5hmC, BRIGHT-seq provides a direct readout based on distinct C-to-A and C-to-T transition signatures, thereby reducing the need for differential subtraction or separate library preparations. This feature enables simultaneous analysis of 5mC and 5hmC within the same sequencing library and can provide information on their co-occurrence within individual DNA molecules. Such single-molecule information may be useful for studying heterogeneous methylation landscapes, allele-specific modifications and epigenetic inheritance mechanisms.

We applied BRIGHT-seq to profile dynamic cytosine modification changes during the differentiation of hESCs and mESCs. In both mESC and hESC, CpG methylation levels increased across diverse genomic features. In hESC, 5hmCpG levels showed a remarkable elevation only within CpG shores, whereas in mESC, prominent increases of 5hmCpG rate were observed universally. Functional annotation indicated that differential (hydroxy)methylated regions are mainly associated with transcriptional regulation. Motif enrichment analyses further revealed that hyper-DMRs were enriched for binding motifs of pluripotency-associated TFs. Hyper-DhMRs were enriched for TF motifs associated with repression of pluripotency and promotion of lineage commitment. Moreover, BRIGHT-seq allowed us to resolve modification states at single-base resolution in gene bodies and promoters of key pluripotency genes, revealing coordinated increases in both 5mC and 5hmC. These findings suggest that dynamic cytosine modifications may play regulatory roles in the transcription of core TF genes during ESC differentiation.

Several limitations of the current BRIGHT-seq workflow should be considered. First, quantitative accuracy remains limited by sequence-context-dependent conversion efficiency and detectable background signals. In particular, inefficient C-to-A conversion in certain AP sequence contexts may lead to underestimation of 5mC levels at corresponding motifs, while cross-calling and quantitative deviations in synthetic dsDNA mixtures indicate that exact absolute quantification, especially at low modification levels or mixed 5mC/5hmC states, remains challenging. In addition, because the current workflow does not include prior blocking of endogenous 5fC and 5caC, these oxidized cytosine derivatives could potentially contribute to 5hmC or 5mC signals, although their impact is expected to be limited due to their low abundance relative to 5mC and 5hmC [32]. Second, site-level quantitative concordance and DNA integrity remain areas for further improvement. Single-CpG-level comparisons showed lower concordance than large-region analyses, suggesting that individual-site quantification should be interpreted cautiously, particularly when per-site read coverage differs across libraries. The 5mC labeling process also caused moderate but non-negligible DNA degradation under our experimental conditions, which may limit applications involving low-input samples. Future optimization will focus on improving conversion efficiency, reducing sequence-context dependence and background signals, incorporating selective blocking strategies and developing milder reaction conditions to improve quantitative accuracy, DNA integrity and low-input compatibility. Although this study focused on bulk population analysis, future adaptation of BRIGHT-seq to single-cell applications would require further optimization but could eventually enable interrogation of epigenetic heterogeneity in complex tissues and disease contexts.

In conclusion, BRIGHT-seq represents a promising strategy for simultaneous mapping of 5mC and 5hmC at single-base resolution. By assigning 5mC and 5hmC to distinct mutation signatures within the same library, BRIGHT-seq offers a direct framework for integrated analysis of these two epigenetic marks. At the same time, further optimization will be needed to improve sequence-context-independent conversion, quantitative accuracy, DNA integrity and low-input compatibility. With these improvements, BRIGHT-seq may become a useful tool for studying the dynamics of DNA modifications in development and disease.

## MATERIALS AND METHODS

Detailed materials and methods are available in the supplementary data.

### Ethical statements

This study met the criteria for exemption from ethical review under Article 32 of the Measures for Ethical Review of Life Science and Medical Research Involving Humans issued under Guo Wei Ke Jiao Fa [2023] No. 4.

## Supplementary Material

nwag353_Supplemental_File

## Data Availability

The raw sequencing data generated in this study have been deposited in the Genome Sequence Archive (GSA) in National Genomics Data Center [32,33] and China National Center for Bioinformation/Beijing Institute of Genomics, Chinese Academy of Sciences (GSA: CRA020813 and HRA009489) that are publicly accessible at https://ngdc.cncb.ac.cn/gsa. Codes for data analysis of BRIGHT-seq are available on GitHub (https://github.com/xinnnluuu/sci_bioinformatics_script/tree/main/BRIGHT-seq_script). All data are available in the main text or the supplementary materials.
