# Supplementary material for: Simultaneous single-base resolution profiling of 5mC and 5hmC using BRIGHT-seq
Source: Natl Sci Rev. 2026 Jun 10;13(14):nwag353. doi: 10.1093/nsr/nwag353 (PMC13386502; doi:10.1093/nsr/nwag353)
Supplement: nwag353_Supplemental_File [file nwag353_supplemental_file.pdf]

Supporting Information

Simultaneous single-base resolution profiling of 5mC and 5hmC using BRIGHT-seq

Xiaochen Xue[1,†], Ziang Lu[1,†], Wei Yang[1,†], Shaoqing Han[1], Zhiying Wang[1], Yifan Jin[1], Xingxing Li[2], Xiang Zhou[1,*], Yafen Wang[3,*], Xiaocheng Weng[1,*]

[1]College of Chemistry and Molecular Sciences, State Key Laboratory of Metabolism and Regulation in Complex Organisms, Taikang Center for Life and Medical Sciences, Wuhan University, Wuhan 430072, China

[2]Institute of Stem Cell and Regeneration, Chinese Academy of Sciences, Beijing 100101, China

[3]School of Public Health, Wuhan University, Wuhan 430072, China

[†]Contributed equally to this work

*Corresponding authors. E-mail: xzhou@whu.edu.cn; yfwang@whu.edu.cn; xcweng@whu.edu.cn

This file includes:

Materials and Methods

Figs S1 to S22

Tables S1 to S3

References

Materials and Methods

Synthesis of A-Ha

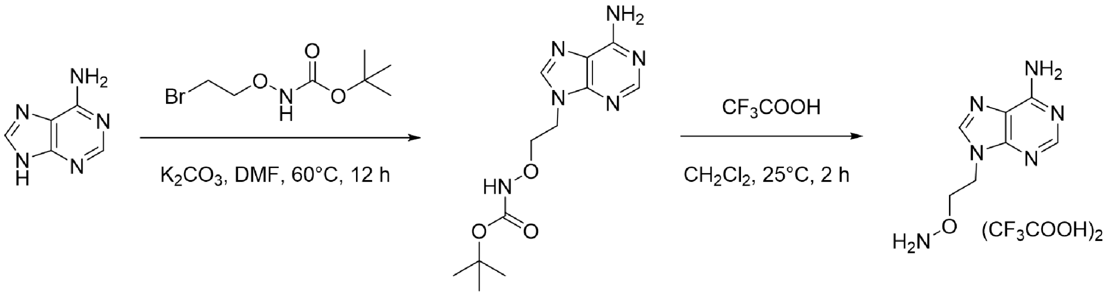

Adenine (1.35 g, 10 mmol), tert-butyl(2-bromoethoxy)carbamate (2.40 g, 10 mmol) and potassium carbonate (1.66 g, 12 mmol) were dissolved in 100 mL N,N-dimethylformamide at 40 °C for 12 h. Then, the reaction mixture was evaporated under reduced pressure and the residue was purified by silica gel chromatography, eluting with a 30:1 dichloromethane / methanol (v/v) to afford A-Boc as a solid (600 mg, yield 20%). ^{1}H NMR (400 MHz, DMSO-d_6): δ 9.53 (s, 1H), 8.14 (s, 1H), 7.98 (s, 1H), 7.19 (s, 2H), 4.29 (t, J = 5.4 Hz, 2H), 3.78 (t, J = 5.4 Hz, 2H), 1.04 (s, 9H). ^{13}C NMR (101 MHz, DMSO-d_6): δ 156.0, 154.9, 152.2, 149.5, 141.1, 118.8, 79.4, 54.9, 49.4, 27.5. HR MS (ESI+): m/z $C_{12}H_{19}N_6O_3^+$ $[M+H]^+$ calculated 295.1513, found 295.1505.

A-Boc (7.4 mg) and 0.5 mL trifluoroacetic acid (TFA) were dissolved in 3.5 mL dichloromethane at room temperature for 2 h. Then, the reaction mixture was evaporated under reduced pressure. Finally, the product was dried under a vacuum for more than 72 hours to afford trifluoroacetate salt of A-Ha as a solid (10.4 mg). ^{1}H NMR (400 MHz, D_2O): δ 8.43 (s, 1H), 8.38 (s, 1H), 4.81 (m, 2H), 3.87 (t, J = 6.0 Hz, 2H). ^{13}C NMR (101 MHz, D_2O): δ 162.8 (q, J = 35.7 Hz), 149.8, 148.8, 144.9, 144.5, 118.3, 116.2 (q, J = 291.3 Hz), 48.9, 38.6. The ratio of A-Ha / TFA (n/n) was quantified as 1:2 by quantitative ^{13}C NMR (800 MHz, D_2O, fluorine decoupling). HR MS (ESI+): m/z $C_7H_{11}N_6O^+$ $[M+H]^+$ calculated 195.0989, found 195.0975.

Oligonucleotide and model DNA preparation

Long single-stranded model DNA containing single 5hmC or 5caC, as well as long single-stranded model DNA with both single 5mC and single 5hmC, were obtained

from Accurate Biology Co., Ltd. (Changsha, China). Short oligonucleotide and long single-stranded model containing one 5mC were purchased from Sangon Biotech Co., Ltd. (Shanghai, China). Complementary strands of the aforementioned models, oligonucleotides containing dU, as well as PCR primers for model DNAs and P7 primer, were also purchased from Sangon Biotech Co., Ltd. (Shanghai, China). Model DNA duplexes were prepared through annealing. In particular, 5'-HEX labeled 80 bp duplex ds-ODN9-mC with single 5mC was PCR-amplified using template ss-ODN9-F (Table S1), Model-1-F and Model-1-R (Table S3) from Sangon Biotech Co., Ltd. (Shanghai, China). In brief, PCR reaction was prepared in 50 μL of 1× DreamTaq Buffer (Thermo Fisher Scientific) with 2 μL of Model-1-F (10 μM), 2 μL of Model-1-R (10 μM), 2 μL of template ss-ODN9-F (100 nM), 0.2 mM of dATP (Invitrogen, 55082), 0.2 mM of dGTP (Invitrogen, 55084), 0.2 mM of dTTP (Invitrogen, 55085), 0.2 mM of 5-methyl-dCTP (NEB, N0356S) and 2.5 units of DreamTaq DNA polymerase (Thermo Fisher Scientific, EP0701). PCR amplification was processed according to the manufacturer's protocols and purified by DNA Clean & Concentrator (Zymo Research).

Cell lines and genomic DNA isolation

Wild-type HEK293T and HeLa cells were cultured in DMEM complemented with 10% fetal bovine serum (FBS) at 37 °C. Wild-type K562 cells were cultured at 37 °C using IMDM contaning 10% FBS. Adriamycin-resistant K562/ADR cells were cultured by supplementing IMDM with 1 μg/mL adriamycin and 10% FBS at 37 °C. These cell lines were purchased from Wuhan Pricella Biotechnology Co., Ltd..

The mESCs, originally bought from the American Type Culture Collection, were cultured at 37 °C on plates coated by 0.1% gelatin in high glucose DMEM, supplemented with 0.1 mM nonessential amino acids, 2 mM L-glutamine, 0.1 mM *β*-mercaptoethanol, 1 mM sodium pyruvate, 1 μM PD0325901 and 3 μM CHIR99021, 1000 U/mL leukemia inhibitory factor (LIF) and 20% FBS. For EBs differentiation, mESCs were cultured in low-attachment dishes at a density of 5×10^5 cells/mL in mESC medium lacking LIF, PD0325901, and CHIR99021. After 2 days, suspension-cultured cells were transferred to 0.2% gelatin-coated dishes and cultured in mESC

medium for further differentiation. Media were changed every other day, and contracting cell patches were collected on 7 days.

The human embryonic stem cell line H9, originally derived from Wicell Research Institute, was cultured in mTeSR™ medium on plates coated by Matrigel. Endoderm differentiation cells was performed following previously reported protocols [1]. For differentiation, DMEM was applied as the basal medium, complemented with 0.2% BSA, 1% penicillin–streptomycin and 1% B27 (without Vitamin A). On day 1, the medium was supplemented with 100 ng/mL Activin A and 2.5 μM CHIR99021, followed by 100 ng/mL Activin A alone for the subsequent 3 days. The differentiated cells were termed as DE cells.

Genomic DNAs (gDNAs) from the aforementioned cells were extracted applying FastPure® Blood/Cell/Tissue/Bacteria DNA Isolation Mini Kit (Vazyme Biotech Co., Ltd, DC112) following the manufacturer's protocol.

Labeling of Oligonucleotide AP Sites with A-Ha

To generate DNA strands containing AP sites, all of the oligonucleotides with dU site were treated with UDG (NEB, M0280S). In a 10 μL reaction, 5 μL of oligonucleotide (100 μM, Table S1) was incubated with 5 units of UDG in 1× UDG reaction buffer at 37 °C for 6 h. The reaction product was extracted by Oligo Clean & Concentrator (Zymo research) or ethanol precipitation in the presence of glycogen (Thermo Fisher Scientific, R0561).

The precipitated DNA was then subjected to A-Ha labeling. The oligo product was treated with 1 mM A-Ha and 40 mM sodium cyanoborohydride ($NaCNBH_3$) (Meryer, M12195-5G) in NaOAc buffer (100 mM, pH 5.0), NH_4OAc buffer (100 mM, pH 4.5) and MES buffer (50 mM, pH 5.0). The reactions were incubated at 37 °C for 6 h in a thermomixer (Thermo Fisher Scientific) at 850 rpm. The labeling reactions were stopped by ethanol precipitation with glycogen and subsequently confirmed by denaturing polyacrylamide gel electrophoresis (PAGE) and mass spectrometry analysis.

Recombinant hTDGcd expression and activity assay

The hTDGcd (amino acids 82-308) with N-terminally hexahistidine-tag was expressed as previously reported [2]. Glycosylase and lyase activity assays of hTDGcd were performed using 5'-HEX labeled 80 bp model dsDNA with single 5caC site (ds-ODN9-caC, Table S1). In brief, 5'-HEX-labeled ds-ODN9-caC was mixed with 66.7 μM hTDGcd in 1× TDG reaction buffer and incubated at 37 °C for 2 h. The excision reaction was purified by DNA Clean & Concentrator kit. The purified DNA was subjected to the reaction with A-Ha following the procedure described above and similarly purified. The tagged product, untreated DNA, and excised AP product were incubated with 5 units of apurinic/apyrimidinic endonuclease 1 (APE 1; NEB, M0282S) at 37 °C for 1 h in 1× NEBuffer 4 (NEB, B7004S). Reactions were terminated by adding 25 μL of formamide, and the mixtures were loaded onto denaturing PAGE for analysis.

5mC labeling, restriction enzyme digestion and Sanger sequencing assay

For 27 bp or 5'-HEX labeled 80 bp dsDNA containing single 5mC (ds-ODN10-mC and ds-ODN9-mC, Table S1), oxidation of TET2 was performed using EpiArt DNA Enzymatic Methylation Kit (Vazyme Biotech Co., Ltd, EM301). The oxidized DNA was processed with hTDGcd for excision, followed by A-Ha labeling as described above, and extracted with DNA Clean & Concentrator kit. The reaction efficiency between ds-ODN9-AP and A-Ha was assessed by denaturing PAGE, and the results for ds-ODN10-mC were further confirmed by MALDI-TOF mass spectrometry.

After spiked with the model duplex containing single 5mC site (ds-ODN12-mC, Table S1), fragmented gDNA was treated with TET2 according to the described protocols. The oxidated product was treated with hTDGcd and labeled with A-Ha, followed by aforementioned purification. The labeled spike-in was amplified using primers Model-3-F and Model-3-R (Table S3) with MightyAmp DNA Polymerase Ver.3 (Takara, R076A). The PCR product was subjected to Sanger sequencing with unified primers by Beijing Tsingke Biotech Co., Ltd. (Beijing, China). For restriction enzyme digestion assay, the PCR product was digested with TaqI-v2 (NEB, R0149V) in 1× rCutSmart buffer (NEB, B6004V) for 1 h at 65 °C, and the fragments were analyzed by agarose gel electrophoresis.

5hmC labeling, restriction enzyme digestion and Sanger sequencing assay

After spiked with the model duplex containing single 5hmC site (ds-ODN11-hmC, Table S1), fragmented gDNA was oxidized by 50 mM $ACT^{+}BF_4^{-}$ (TCI, A2065) in sodium phosphate buffer (50 mM, pH 7.5) at 37 °C for 4 h. The reaction was then complemented to a final concentration of 100 mM sodium phosphate (pH 7.5). The oxidized DNA was purified using 1.8× VAHTS DNA Clean Beads (Vazyme Biotech Co., Ltd., N411). The oxidation product was then subjected to a reaction containing 150 mM malononitrile (J&K, 261700) in either H_2O, 10 mM NH_4OAc buffer (pH 7.0) or 10 mM Tris-HCl buffer (pH 7.0) at 37 °C for 24 h with shaking at 850 rpm. The tagged DNA was extracted using DNA Clean & Concentrator kit. The spike-in was amplified using with MightyAmp DNA Polymerase Ver.3, Model-2-F and Model-2-R primers (Table S3). The PCR products were subjected to Sanger sequencing as previously described. For restriction enzyme digestion assay, the PCR products were digested with BamHI-HF (NEB, R3136T) in 1× rCutSmart buffer at 37 °C for 1 h, and the resulting fragments were analyzed by agarose gel electrophoresis.

Enzymatic digestion and mass spectrometry analysis

Oligonucleotides, including ODN1-dU and ds-ODN10-mC, were prepared and chemically labeled as described above. These oligonucleotides were characterized by MALDI-TOF (Shimadzu, Japan) or ESI-MS (Sangon Biotech Co., Ltd.) in negative mode.

For enzymatic digestion of short oligonucleotides, each DNA was denatured by heating for 5 min at 95 °C and then immediately chilling on ice for at least 3 min. The denatured DNA was incubated with S1 nuclease (Takara, 2410A) in 1× S1 nuclease buffer (Takara) at 37 °C for at least 8 h. The mixture was then treated with alkaline phosphatase (Calf intestine) (Takara, 2250A) and venom phosphodiesterase I (Sigma-Aldrich, P3134-100MG) in 1× alkaline phosphatase buffer (Takara) at 37 °C for additional 8 h. For fragmented H9 gDNA, 1 μL Stop reagent were added to the oxidation mixture to terminate TET2 oxidation at 37 °C for 30 min before purification. The enzymatic digestion reactions were performed as mentioned above.

HPLC–MS analysis of the digested DNA was performed on Thermo Fisher Scientific LTQ Orbitrap Elite mass spectrometer in positive ESI mode, coupled with an XD8-C18 column (1.8 μm, 2.1 mm × 100 mm, Agilent) maintained at 25 °C. The mobile phase was composed of A (water containing 0.1% formic acid, v/v) and B (methanol containing 0.1% formic acid, v/v), with a flow rate of 0.2 mL/min. Nucleosides were eluted using the following gradient: 3% B to 15% B over 5 min, 15% B to 50% B over 3 min, 50% B to 3% B over 1 min, and 3% B for 6 min.

HPLC–MS/MS analysis of the digested DNA was performed on a triple quadrupole mass spectrometer (QTRAP5500+, ABSciex) in positive ESI mode, coupled with a Shim-pack GIST C18 column (2.0 μm, 2.1 mm × 100 mm, Shimadzu) maintained at 30 °C. The mobile phase was composed of A (water containing 0.1% formic acid, v/v) and B (methanol containing 0.1% formic acid, v/v), with a flow rate of 0.2 mL/min. Nucleosides were eluted using the following gradient: 5% B for 3 min, 5% B to 80% B over 7 min, 80% B for 3 min, 80% B to 5% B over 30 s and 5% B for 6.5 min. The source-dependent parameters were as follows: curtain, 30 psi; nebulizer gas, 55 psi; turbo heater gas, 55 psi; ion spray voltage, 5500 V; source temperature, 550 °C. Mass transitions (precursor ions → product ions) were dA (252.1 → 136.1), dG (268.0 → 152.1), dC (228.1 → 112.0), dT (243.0 → 127.0), 5mC (242.1 → 126.1), 5hmC (258.2 → 142.1), 5fC (256.1 → 140.0) and 5caC (272.1 → 156.0). Nucleoside concentrations in each DNA sample were determined by fitting the peak areas to standard curves.

Potential degradation tests of 5mC labeling steps

A 116 bp 5mC-containing model dsDNA (ds-ODN13-mC, Table S1), fragmented or unfragmented gDNA from K562 cells was subjected to the 5mC labeling reactions as described above. For the model DNA, 100 pg of purified product after each step was analyzed by qPCR using the primers Model-3-F and Model-3-R and MightyAmp DNA Polymerase Ver.3. For gDNA, agarose gel electrophoresis and TapeStation electrophoresis were performed on the products from unfragmented and fragmented gDNA collected after each step, respectively.

Sequence bias tests of A-Ha labeling and PCR amplification

A 104 bp synthetic dsDNA containing a single dU site flanked by randomized bases (NN-dU-NN, N = A, T, C, or G) was excised by UDG and then treated with A-Ha. The labeled products were further subjected to end repair and ligation with NEBNext Adaptor for Illumina (NEB) using the VAHTS Universal DNA Library Prep Kit for Illumina V3 (Vazyme Biotech Co., Ltd., ND607) following the manufacturer's protocol. MightyAmp DNA Polymerase Ver.3 was used for one round of primer extension with NEBNext Index Primer for Illumina (NEB). The extension products were incubated with lambda exonuclease (NEB, M0262S) to remove 5'-phosphorylated templates and purified with 0.9× VAHTS DNA Clean Beads. The purified samples were PCR-amplified using NEBNext Ultra II Q5 Master Mix (NEB, M0544), P7 primer (Table S3), and NEBNext Universal PCR Primer for Illumina (NEB), following the manufacturer's protocol. The resulting PCR products were purified using 0.9× VAHTS DNA Clean Beads. The libraries were sequenced on an Illumina NovaSeq 6000 platform (GENEWIZ, China).

BRIGHT-seq library construction for simultaneous detection of genomic 5mC and 5hmC

0.03-3 μg gDNA spiked with unmethylated λDNA (0.1%, Thermo Fisher Scientific) was sonicated into fragments approximately 200 bp in size using Bioruptor Plus sonication device (Diagenode). The fragmented DNA mixture, mixed with a 116 bp duplex spike-in containing 5mC and 5hmC (ds-ODN13-mChmC, 0.1%, Table S1), was subjected to end repair and ligation with NEBNext Adaptor for Illumina using VAHTS Universal DNA Library Prep Kit for Illumina V3 following the manufacturer's protocols. The ligated DNA was first treated with $ACT^+BF_4^-$ for oxidation and subsequently labeled with malononitrile in H_2O as described previously. The product was further oxidized using TET2, followed by excision with hTDGcd according to the previously described method. The resulting DNA was treated with proteinase K (NEB, P8107S) to digest proteins and purified using 1.2× VAHTS DNA Clean Beads. Finally, the purified DNA was labeled with A-Ha. MightyAmp DNA Polymerase Ver.3 was used

for one round primer extension with NEBNext Index Primer for Illumina. The extension products were incubated with lambda exonuclease to remove 5'-phosphorylated templates and purified with 0.9× VAHTS DNA Clean Beads. The purified samples were PCR-amplified using the NEBNext Ultra II Q5 Master Mix, P7 primer (Table S3), and NEBNext Universal PCR Primer for Illumina following the manufacturer's protocol. The resulting PCR products were purified using 0.9× VAHTS DNA Clean Beads. The libraries were sequenced on an Illumina NovaSeq 6000 platform (GENEWIZ, China) and MGISEQ-2000 platform (Peking University Chengdu Academy for Advanced Interdisciplinary Biotechnologies, China).

EM-seq library construction

The conversion steps of EM-seq were performed using the standard protocol of EpiArt DNA Enzymatic Methylation Kit with fragmented gDNA spiked with fragmented unmethylated λDNA and ds-ODN13-mChmC (0.1% each). The resulting products were directly used for library preparation and amplification with the EpiArt DNA Methylation Library Kit for Illumina V3 (Vazyme Biotech Co., Ltd., NE103) following the manufacturer's protocols. The libraries were sequenced on an Illumina NovaSeq 6000 platform (GENEWIZ, China).

ACE-seq library construction

The conversion steps of ACE-seq were performed using the standard protocol of EpiArt DNA Enzymatic Methylation Kit with fragmented gDNA spiked with fragmented unmethylated λDNA and ds-ODN13-mChmC (0.1% each). Briefly, mixed DNA sample was glycosylated by β-glucosyltransferase at 37 °C for 2 h without the addition of TET2. Purified glycosylation product was denatured followed by deamination with the treatment of APOBEC3A at 37 °C for 4 h. After purification with 1.8× VAHTS DNA Clean Beads, the resulting products were directly used for library preparation and amplification with the EpiArt DNA Methylation Library Kit for Illumina V3 following the manufacturer's protocols. The libraries were sequenced on an Illumina NovaSeq 6000 platform (Novogene, China).

Sequencing Data Alignment and Mutation Calling

The sequencing data analyzed in this study included BRIGHT-seq, SIMPLE-seq (GSM5929312), TAPSβ (GSM4708553), EM-seq, ACE-seq, and model DNA data [3,4]. Single-cell data from SIMPLE-seq were processed as pseudo-bulk data. Prior to alignment, all raw sequencing reads were processed using Trim Galore (v0.6.7) to remove low-quality bases and adapter sequences.

For the BRIGHT-seq, SIMPLE-seq, and TAPSβ datasets, clean reads were aligned to the hg38 or mm39 reference genomes using hisat-3n (v2.2.1) under either the T-A or T-C mode [5]. Following alignment, read splitting was performed for specific datasets. For BRIGHT-seq, aligned reads were categorized by their strand of origin based on SAM Flag values: reads with a Flag of 96 or 144 were assigned to the positive strand, while those with 80 or 160 were assigned to the negative strand. For SIMPLE-seq, reads carrying 5mC and 5hmC information were separated using the custom scripts provided in the original publication. PCR duplicates for these three data types were subsequently removed using Picard MarkDuplicates (v2.20.4). Mutation sites were then called from the deduplicated reads using REDItools2 with the following filtering thresholds: a mutation rate > 10%, a mutation count ≥ 2, and a total read coverage (mutation count plus reference count) ≥ 3 [6]. Putative mutation sites were further filtered to exclude background SNPs, which were defined as sites present in the BRIGHT-seq control data with a mutation rate > 5% and a mutation count ≥ 5. The final set of modified sites was determined by retaining only the intersection of sites consistently identified across biological replicates.

For EM-seq and ACE-seq datasets, reads were aligned to the hg38 or mm39 genomes using Bismark (v0.23.1) [7]. PCR duplicates were removed using the deduplicate_bismark subcommand, and modification information was extracted using the bismark_methylation_extractor subcommand. Consistent with the previous pipeline, the final modified sites were defined as those exhibiting a mutation rate > 10%, a mutation count ≥ 2, and a total read coverage ≥ 3, provided they were consistently detected across all biological replicates.

Model DNA data were aligned to their corresponding reference sequences using

Bowtie2 (v2.2.5) [8]. Similar to the BRIGHT-seq processing strategy, reads were separated by their strand of origin based on their Flag values. The mutation rates at distinct positions or within random flanking sequence were directly calculated using custom scripts.

Downstream Analysis of Modification Sites

To visualize mutations at specific genomic location, aligned sequencing reads were examined using the Integrative Genomics Viewer (IGV) [9]. To enhance visualization clarity, insertions and deletions (INDELs) shorter than 5 nucleotides were filtered out. To determine the spatial relationship between 5mC and 5hmC, the distance from each 5mC to its nearest 5hmC site was calculated. These distances were categorized based on their sequence context (5mCpG, 5mCHG, and 5mCHH). The proportion of 5mC sites located at distances ranging from 1 to 1000 bp, or greater than 1000 bp, relative to the total 5mC count was calculated. The cumulative proportions were then plotted across the specified distance bins.

Considering that 5mC-derived signals may introduce not only the expected A reads but also spurious T reads, which can bias 5hmC quantification, we implemented a correction strategy. Specifically, the T/A ratio at CpG sites was estimated based on strand-specific patterns derived from flanking random sequence contexts of 5mC sites. In downstream analysis of average modification level, A counts were corrected as $A_{corrected} = A + A * (Ratio_{T/A})$, and T counts were corrected as $T_{corrected} = T - A * (Ratio_{T/A})$, thereby accounting for misincorporation effects introduced by 5mC.

For the analysis of modification site distribution, gene body annotations were obtained from GENCODE (v45), and histone modification regions were sourced from the ENCODE project (files ENCFF559MMQ and ENCFF470HOG) [10-12]. The mutation information was converted to bedGraph format, and corresponding bigWig files were generated. The spatial distribution of these sites across gene bodies, as well as their proximity to histone modification signals, was assessed using the computeMatrix tool from the deepTools package [13].

To evaluate the correlation between samples, the genome was divided into fixed-size

windows. The modification level of the identified sites within each window was calculated, and the Pearson correlation coefficient of the resulting data matrix was computed. For this analysis, only modified cytosine sites were considered, and the modification level at each site was calculated following the REDItools framework, defined as the mutation count divided by the sum of the mutation count and the reference base count. Notably, to isolate the 5mC sites detected by EM-seq, the final modified sites identified in the ACE-seq samples were subtracted from the EM-seq datasets.

To analyze the distribution of modification levels across genomic functional elements, annotation data for promoters, exons, introns, CpG islands, and repeat regions (based on GENCODE v45) were downloaded from the UCSC Table Browser. Promoters were defined as the 1000 bp regions upstream of transcription start sites (TSSs), while CpG shores were defined as the 2000 bp regions immediately upstream and downstream of CpG islands. Enhancer annotations were obtained from EnhancerAtlas, and their genomic coordinates were lifted over to the hg38 and mm39 assemblies where applicable [14]. Using deepTools, these functional regions were scaled into 100 equal-sized bins, and the modification level within each bin was calculated. In this context, all cytosine sites were included, and the average modification level was computed as the ratio of mutation counts to the total counts of all four nucleotides.

Differential Modification Analysis

Differential modification analysis was performed using the methylKit package (v1.26.0) to identify both differentially methylated regions (DMRs) and differentially hydroxymethylated regions (DhMRs) [15]. For this analysis, the genome was tiled into 1000 bp windows. To ensure high-quality quantification, regions were only retained if they contained more than 10 covered CpG sites, with a minimum sequencing coverage of 5 per individual site.

To define statistically significant differential regions, the false discovery rate (FDR) was controlled, requiring a q value < 0.05. Additionally, the threshold for the absolute

difference in hydroxymethylation levels was set at > 15% for both the human and mouse genomes. For differential methylation, the absolute difference thresholds were set at > 20% for the human genome and > 40% for the mouse genome. The statistically significant results were subsequently visualized using volcano plots.

Genomic annotation of the identified DMRs and DhMRs was conducted using the annotatePeaks.pl script from the HOMER software suite (v4.11), and motif enrichment analysis was performed using the findMotifsGenome.pl script [16]. Gene Ontology (GO) enrichment analysis for the associated genes was carried out using the DAVID online platform [17].

Supplementary Figures

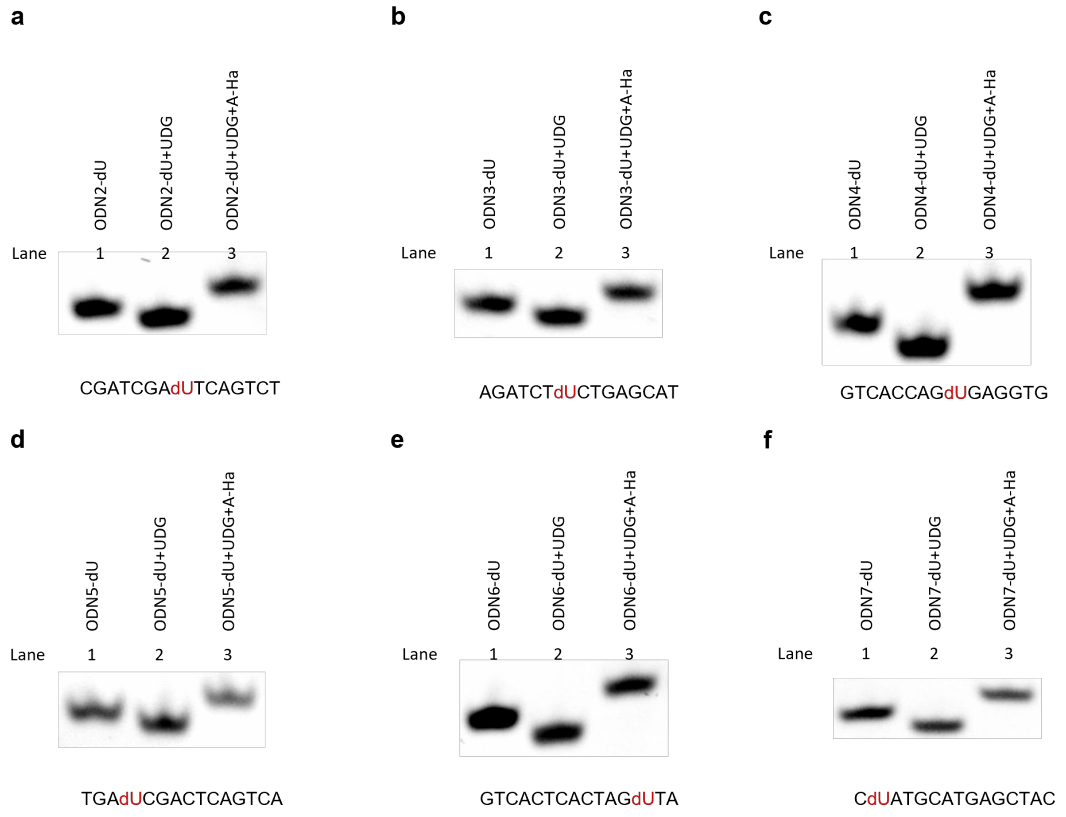

Figure S1. PAGE analysis of A-Ha labeling efficiency for 15-nt model oligonucleotides with different sequences. (a-f) The analysis of ODN2-dU (a), ODN3-dU (b), ODN4-dU (c), ODN5-dU (d), ODN6-dU (e) and ODN7-dU (f), respectively. Lane 1: untreated ODN-dU; lane 2: ODN-dU treated with UDG; lane 3: ODN-AP labeled with A-Ha in 100 mM NaOAc buffer (pH 5.0).

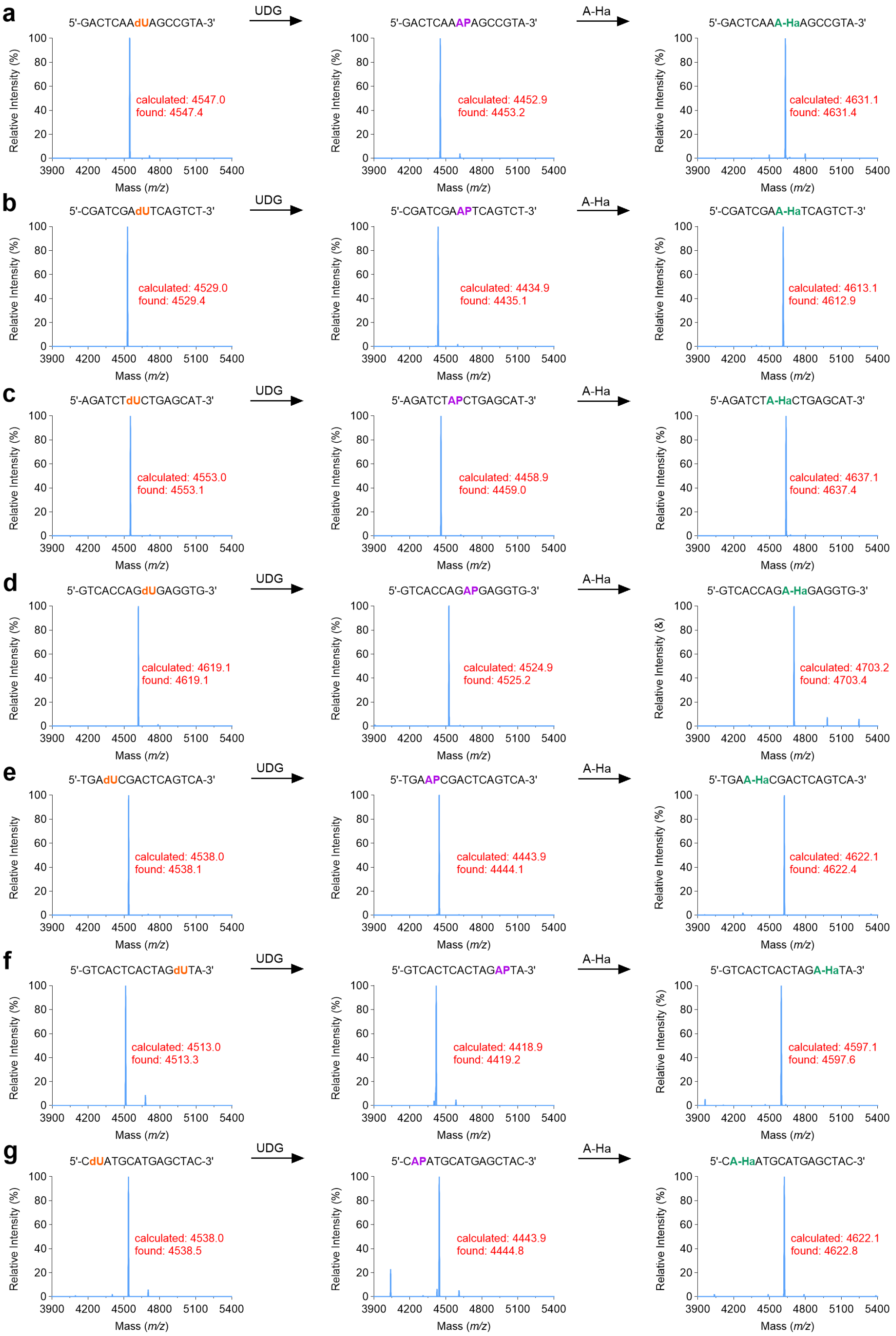

Figure S2. ESI–MS characterization of 15 nt model oligonucleotides with different sequences without treatment, after UDG excision and after A-Ha labeling in NaOAc buffer (100 mM). (a-g) Results of ODN1-dU (a), ODN2-dU (b), ODN3-dU (c), ODN4-dU (d), ODN5-dU (e), ODN6-dU (f) and ODN7-dU (g), respectively.

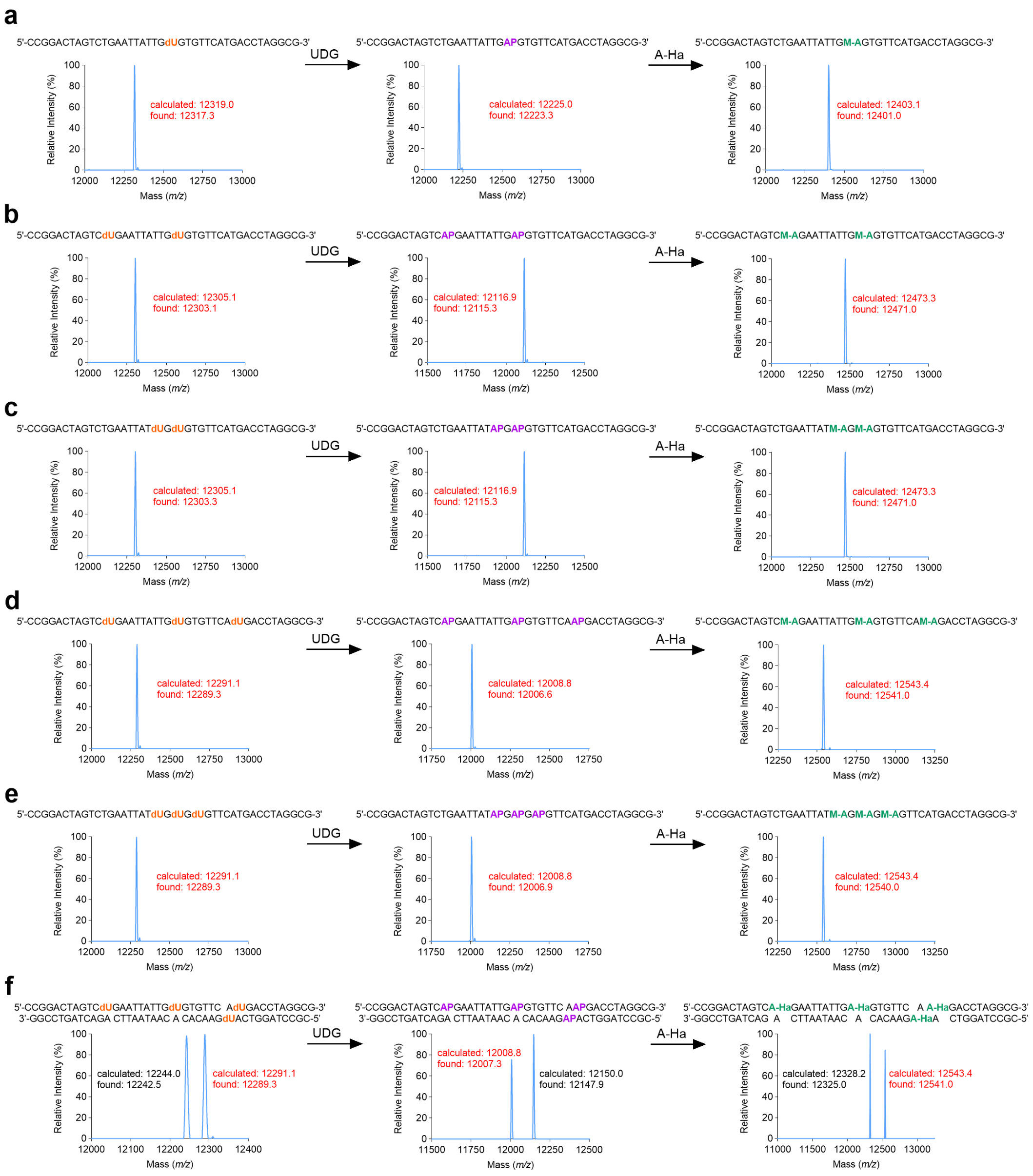

Figure S3. ESI–MS characterization of model oligonucleotides containing different numbers of dU without treatment, after UDG excision, and after A-Ha labeling in NaOAc buffer (100 mM). (a-f) Results of ODN8-1U (a), ODN8-2U (b), ODN8-U2 (c), ODN8-3U (d), ODN8-U3 (e), and ds-ODN8-4U (f), respectively.

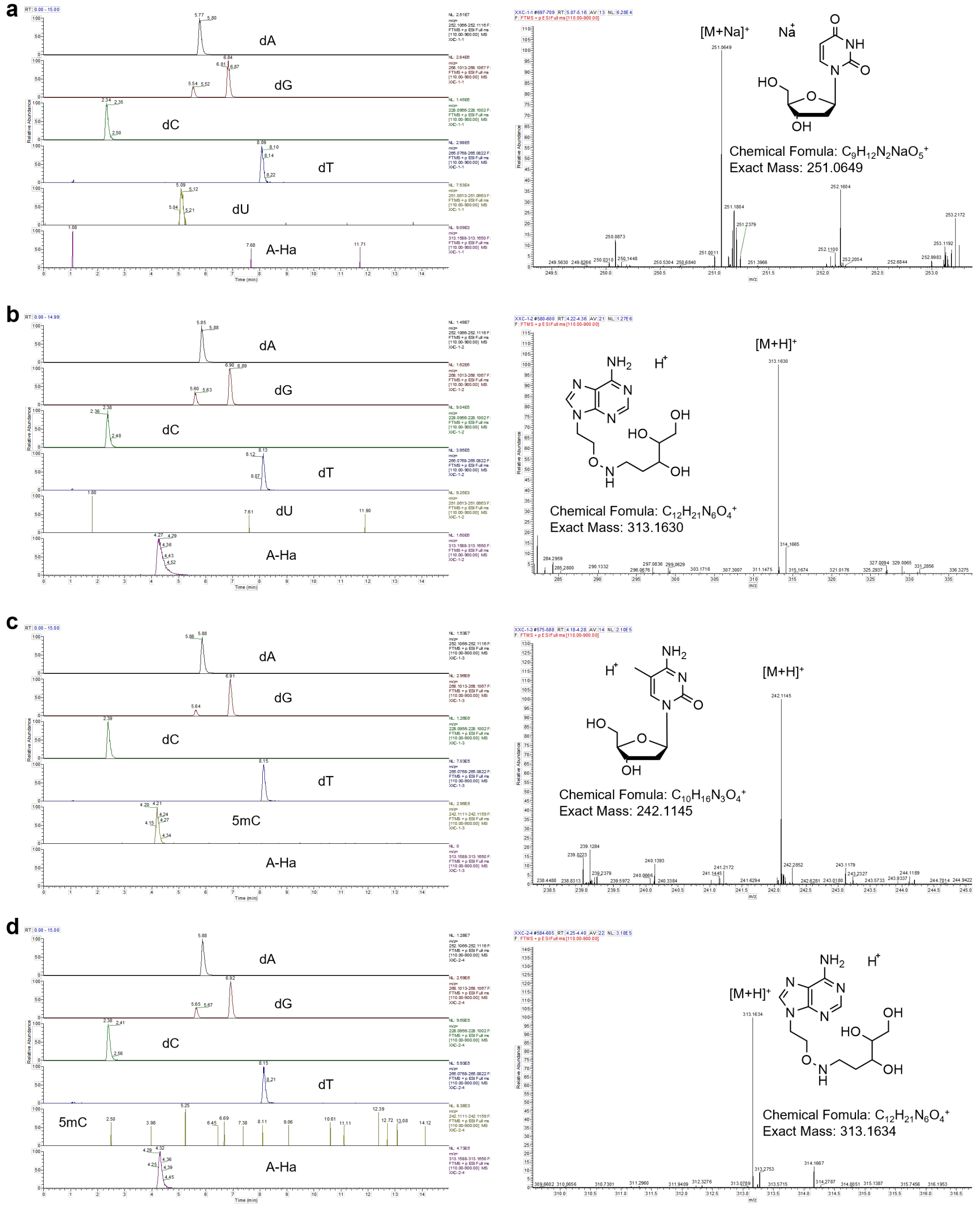

Figure S4. HPLC–MS spectrum of enzymatically digested model DNAs before and after A-Ha labeling. (a) Untreated ODN1-dU. For dU, $[M+Na]^+_{cal}$ = 251.0638, $[M+Na]^+_{obs}$ = 251.0649. (b) ODN1-dU after A-Ha labeling. For A-Ha, $[M+H]^+_{cal}$ = 313.1619, $[M+H]^+_{obs}$ = 313.1630. (c) Untreated ds-ODN10-mC. For 5mC, $[M+H]^+_{cal}$ = 242.1135, $[M+H]^+_{obs}$ = 242.1145. (d) ds-ODN10-mC after A-Ha labeling. For A-Ha, $[M+H]^+_{cal}$ = 313.1619, $[M+H]^+_{obs}$ = 313.1634.

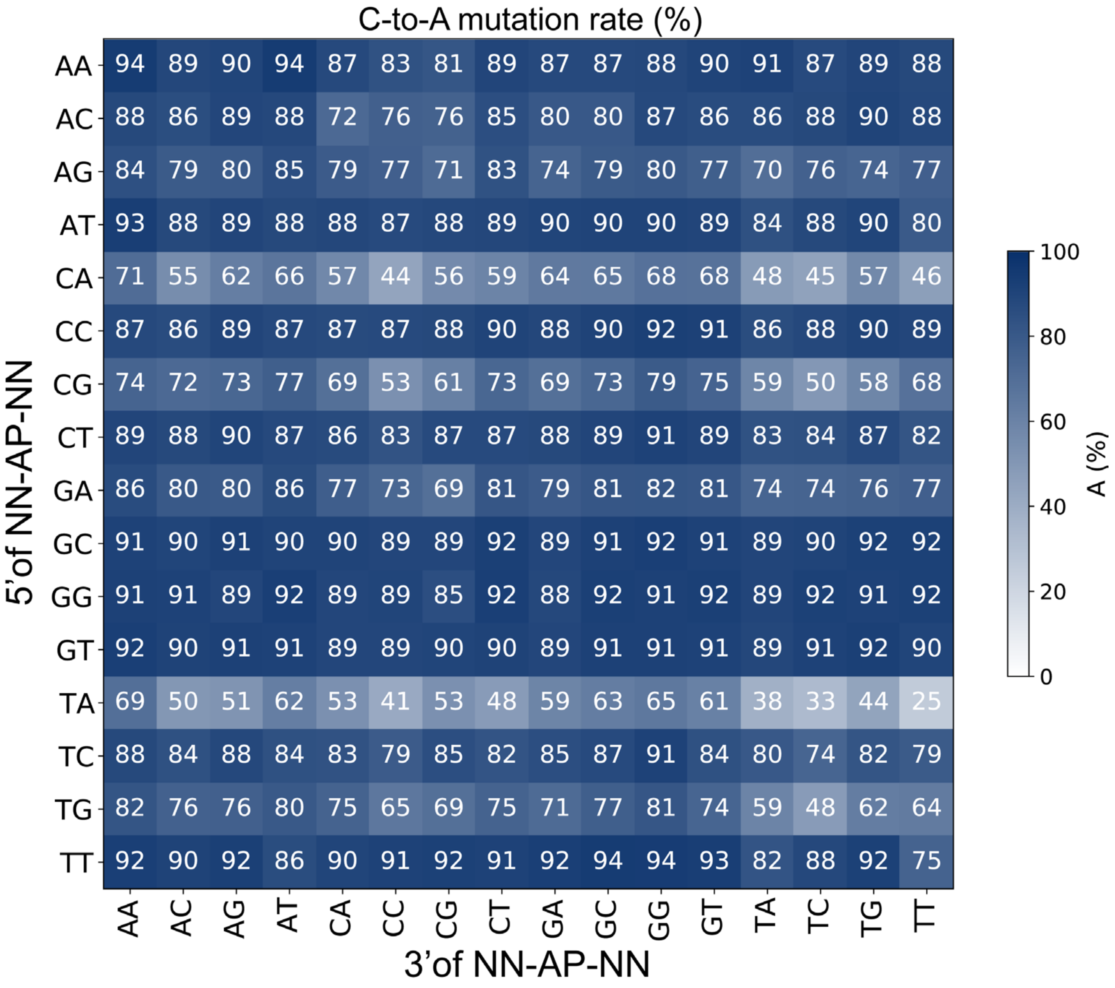

Figure S5. Heatmap plot for mutation ratios on 256 AP motifs (NN-AP-NN) after A-Ha labeling and PCR amplification.

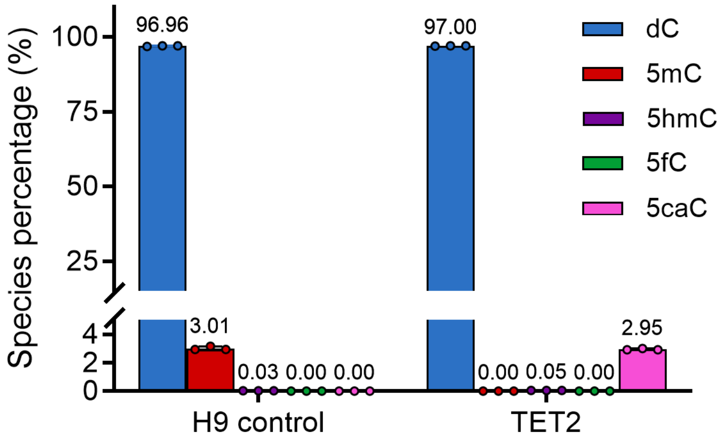

Figure S6. HPLC–MS/MS quantification of the relative percentages of unmodified and modified cytosines in H9 gDNA before and after TET2 oxidation. Data are shown as mean ± s.d. from three independent experiments (n = 3).

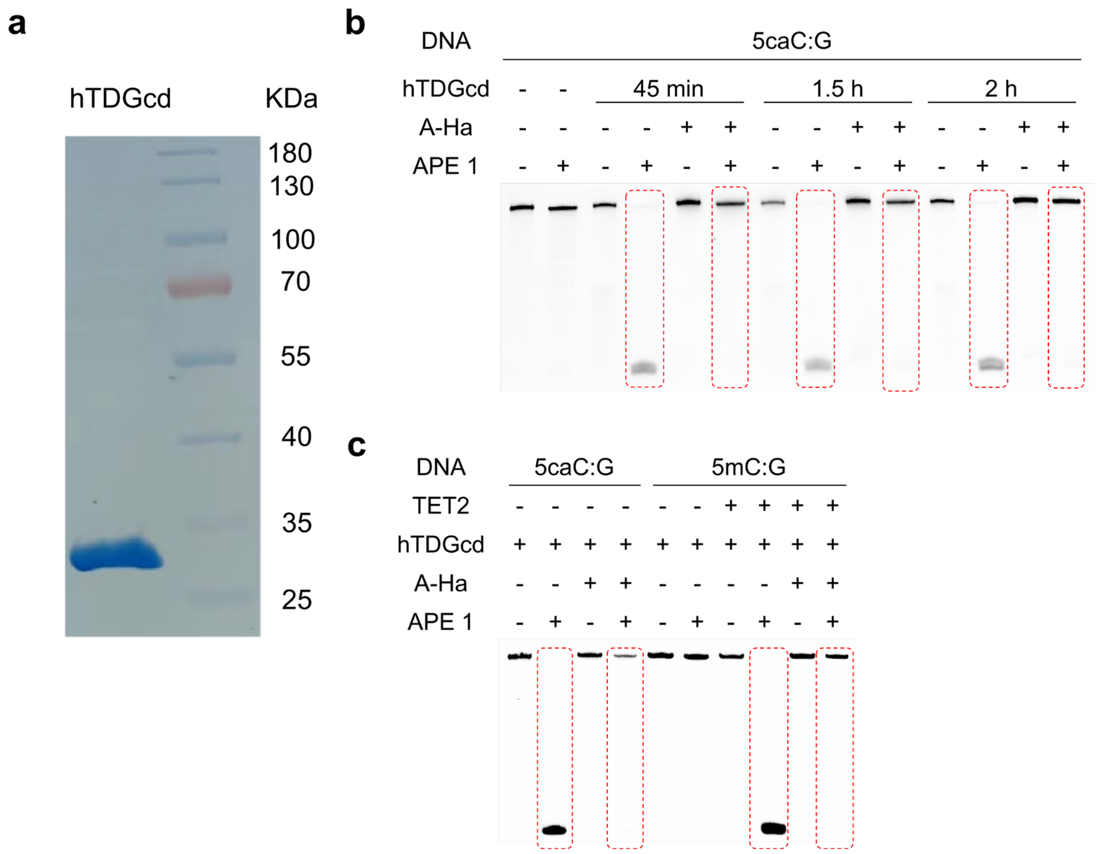

Figure S7. Activity verification of purified hTDGcd and commercial TET2 by gel electrophoresis. (a) SDS-PAGE analysis of purified hTDGcd. (b) PAGE of ds-ODN9-caC without treatment or subjected to hTDGcd excision for different times and further APE 1 cleavage. (c) PAGE of ds-ODN9-mC without treatment or subjected to TET2 oxidation, hTDGcd excision, A-Ha labeling and further APE 1 cleavage. ds-ODN9-caC without treatment or subjected to hTDGcd excision, A-Ha labeling and further APE 1 cleavage were used as controls.

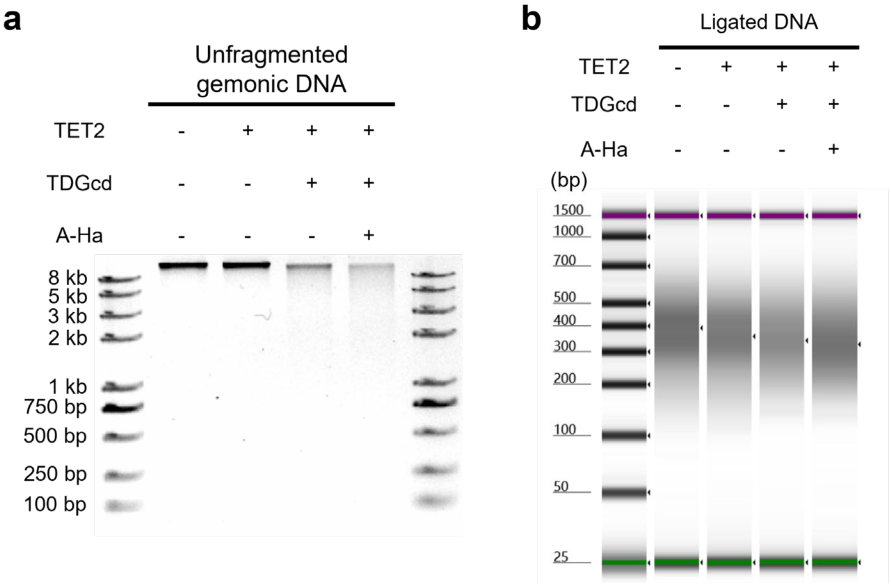

Figure S8. Potential degradation tests of 5mC labeling steps. (a) Agarose gel image of untreated DNA and products from K562 gDNA after treatment steps. (b) TapeStation electropherograms of fragmented, adapter-ligated K562 gDNA after treatment steps.

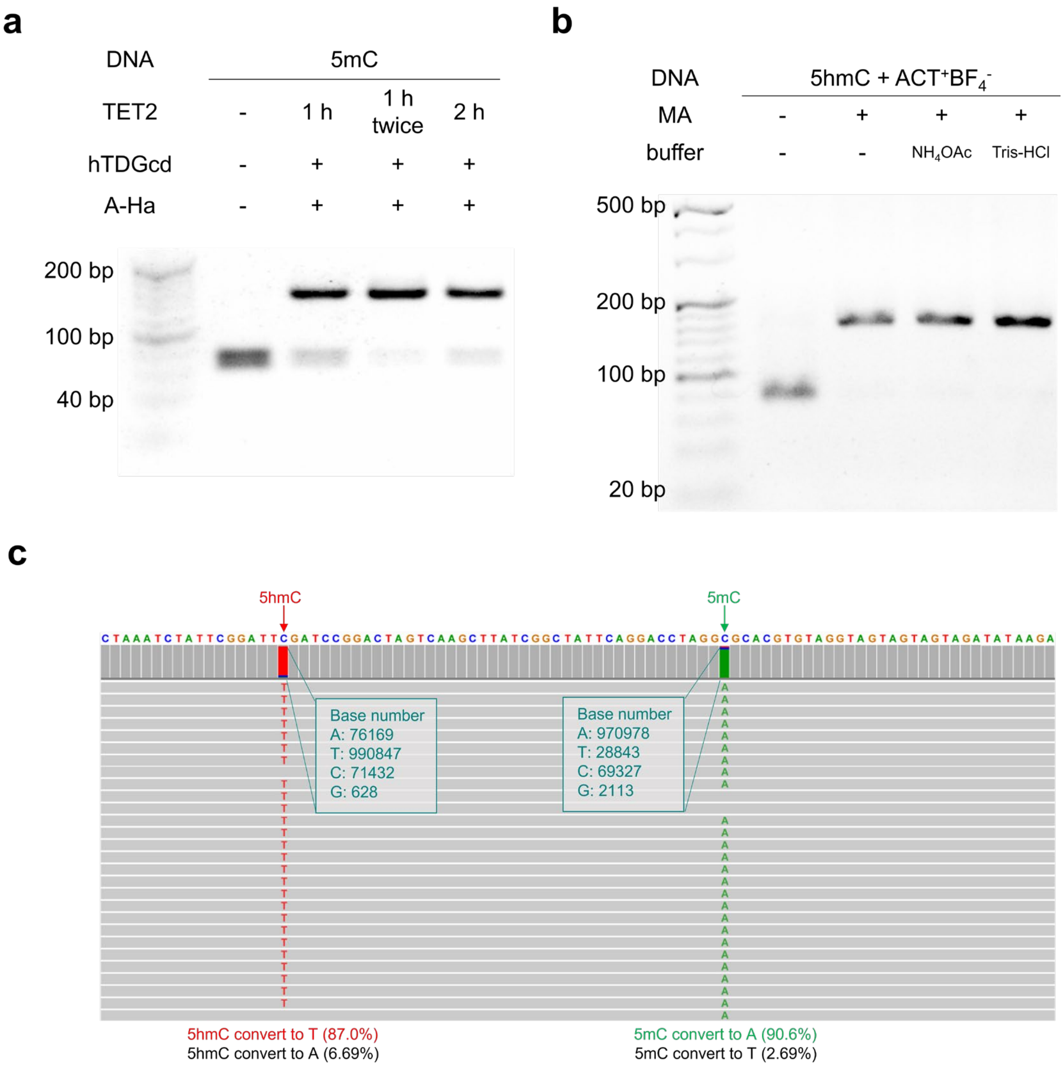

Figure S9. Conversion efficiencies of 5mC-to-A and 5hmC-to-T on model dsDNAs. (a) Validation of 5mC-to-A conversion by TaqI-v2 cleavage. The 80 bp ds-ODN12-mC with single 5mC within single TaqI-v2 site (T^CGA) was either untreated or subjected to TET2 oxidation for varying times, hTDGcd cleavage and A-Ha labeling, followed by PCR amplification. The resulting 138 bp PCR products were incubated with TaqI-v2. (b) Validation of 5hmC-to-T conversion by BamHI-HF cleavage. The 100 bp ds-ODN11-hmC with single 5hmC within single BamHI-HF site (G^GATCC) was either untreated or subjected to $ACT^+BF_4^-$ oxidation and malononitrile labeling with various buffers, followed by PCR amplification. Lane 1: untreated; lane 2: malononitrile reaction in H_2O; lane 3: malononitrile reaction in 10 mM NH_4OAc buffer (pH 7.0); lane 4: malononitrile reaction in 10 mM Tris-HCl buffer (pH 7.0). The 157 bp PCR products were incubated with BamHI-HF. (c) Integrative genomics viewer (IGV) showing the

sequenced reads aligned to spiked model dsDNA containing 5mC and 5hmC (ds-ODN13-mChmC).

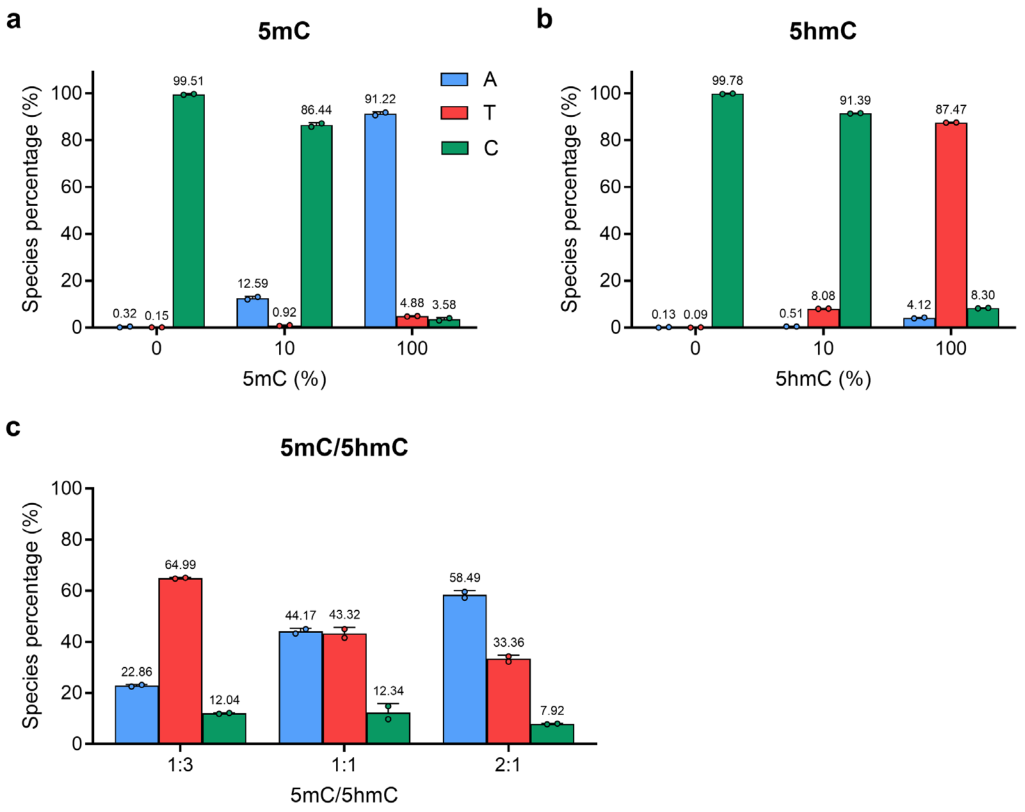

Figure S10. Performance evaluation of BRIGHT-seq using synthetic dsDNA mixtures. (a) Detection of 5mC and false-positive 5hmC signals in synthetic dsDNA mixtures containing defined fractions of 5mC and unmodified C at the same cytosine position. The C-to-A signal represents detected 5mC, whereas the C-to-T signal represents false-positive 5hmC calling. At the 10% input 5mC level, the detected C-to-A signal was 12.59%, and the false-positive 5hmC calling rate was 0.92%. At the 100% input 5mC level, the false-positive 5hmC calling rate was 4.88%. (b) Detection of 5hmC and false-positive 5mC signals in synthetic dsDNA mixtures containing defined fractions of 5hmC and unmodified C at the same cytosine position. The C-to-T signal represents detected 5hmC, whereas the C-to-A signal represents false-positive 5mC calling. At the 10% input 5hmC level, the false-positive 5mC calling rate was 0.51%. At the 100% input 5hmC level, the false-positive 5mC calling rate was 4.12%. (c) Detection of mixed 5mC and 5hmC populations at the same cytosine position in synthetic dsDNA mixtures with defined 5mC:5hmC ratios. For input 5mC:5hmC ratios of 1:3, 1:1, and 2:1, the detected C-to-A/C-to-T signals were 22.86%/64.99%, 44.17%/43.32%, and

58.49%/33.36%, corresponding to detected ratios of approximately 1:2.84, 1:1, and 1.75:1, respectively. Data are shown as mean ± s.d. from two independent experiments (n = 2).

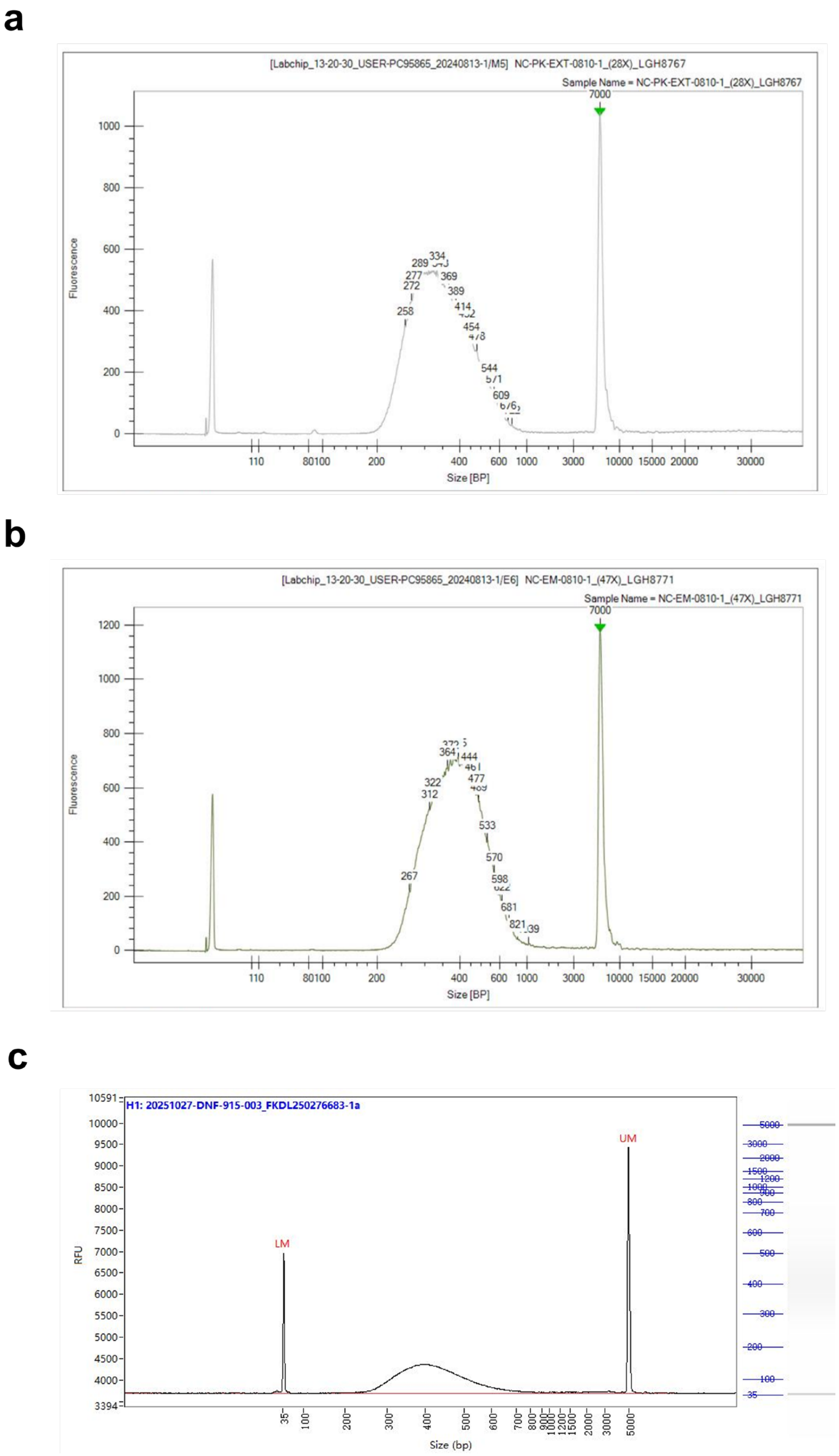

Figure S11. DNA size distribution of sequencing libraries from mESC gDNA. (a) Labchip GX trace of BRIGHT-seq library. (b) Labchip GX trace of EM-seq library. (c) Fragment Analyzer (DNF-915) trace of ACE-seq library. Because these sequencing

libraries were prepared and sequenced by different service providers, the library QC profiles were generated using different instruments: Labchip GX (GENEWIZ, China) for the BRIGHT-seq and EM-seq libraries, and Fragment Analyzer (DNF-915) (Novogene, China) for the ACE-seq library. Although the instruments differ, these QC profiles consistently show the DNA size distributions of the libraries and do not indicate apparent overamplification.

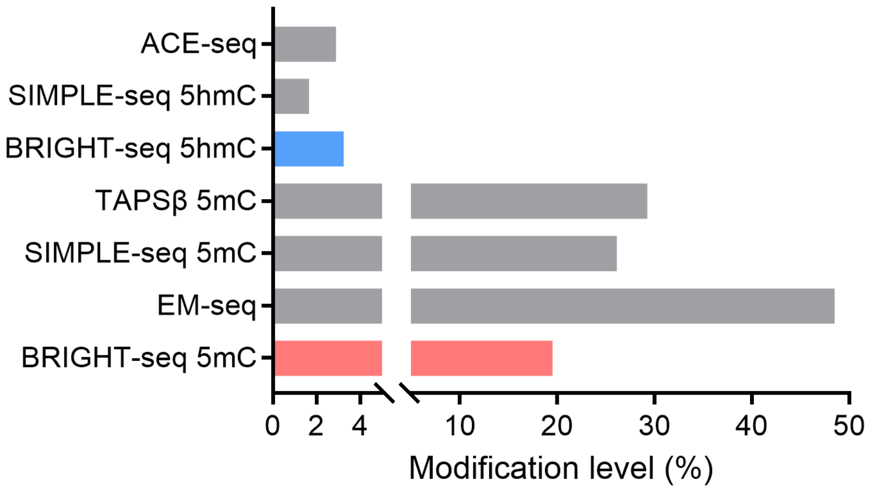

Figure S12. Comparison of average 5mC and 5hmC levels at CpG sites measured by BRIGHT-seq and previously published 5mC/5hmC sequencing methods in gDNA from 2i-cultured mESCs.

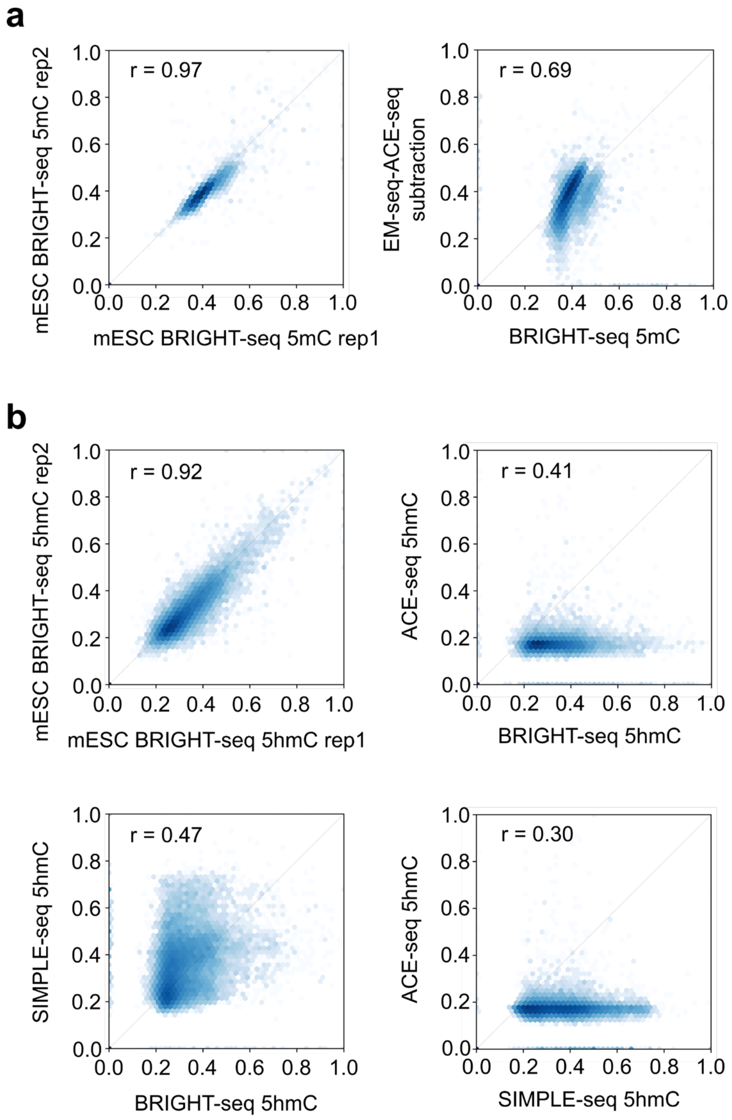

Figure S13. Correlation density plots in mESC gDNA between two replicates of BRIGHT-seq or between BRIGHT-seq and other published 5mC and 5hmC sequencing methods in large regions. (a) Correlation density plots of 5mCpG signals within BRIGHT-seq replicates, and between BRIGHT-seq and EM-seq–ACE-seq subtraction in 10 kb bins. (b) Correlation density plots of 5hmCpG signals within BRIGHT-seq replicates, and between BRIGHT-seq ACE-seq, and SIMPLE-seq in 100 kb bins.

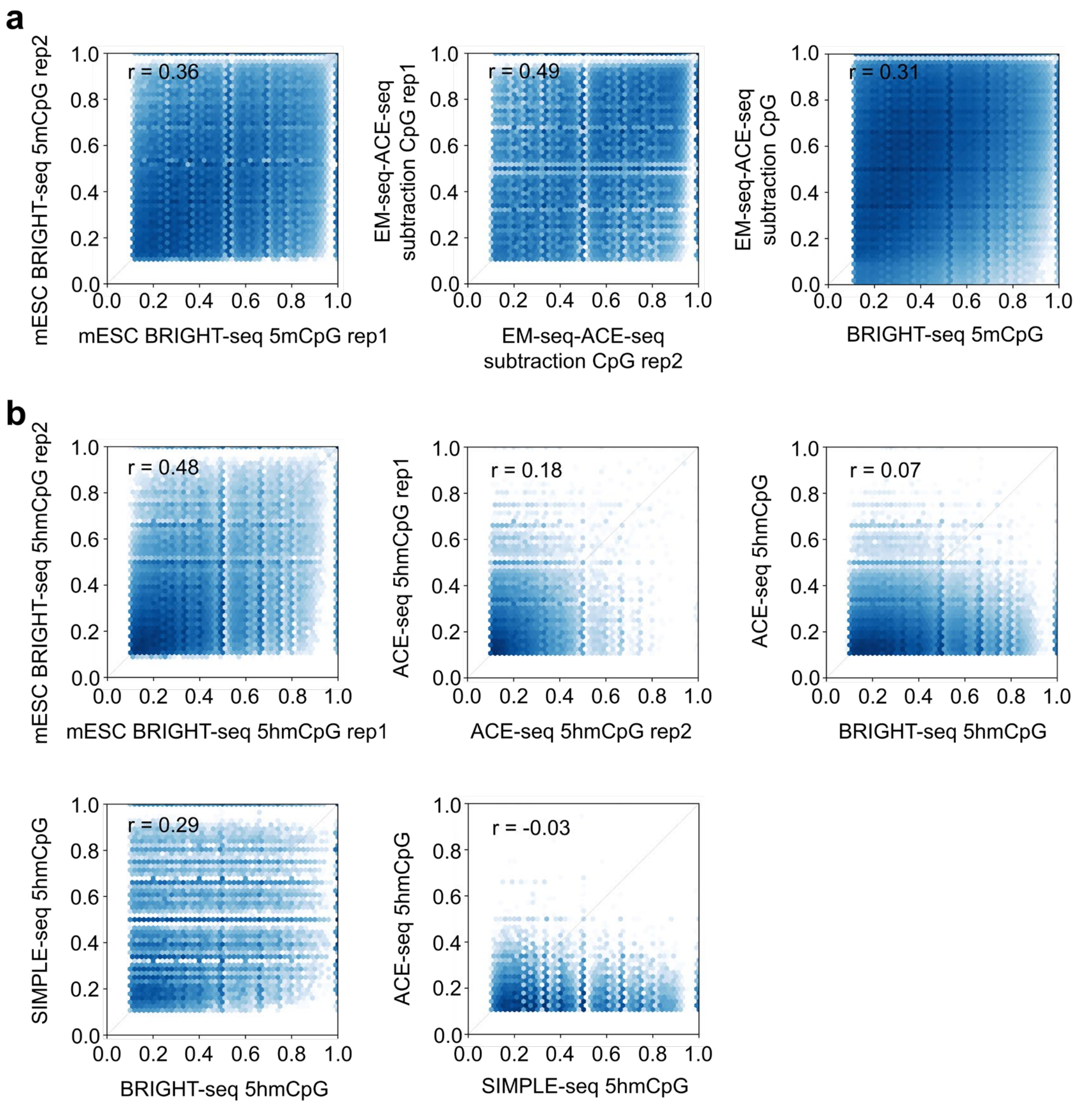

Figure S14. Site-level correlation density plots at shared CpG sites in mESC gDNA. (a) Correlation density plots of 5mCpG signals within BRIGHT-seq replicates, within EM-seq replicates, and between BRIGHT-seq and EM-seq–ACE-seq subtraction. (b) Correlation density plots of 5hmCpG signals within BRIGHT-seq replicates, within ACE-seq replicates, and between BRIGHT-seq, ACE-seq and SIMPLE-seq.

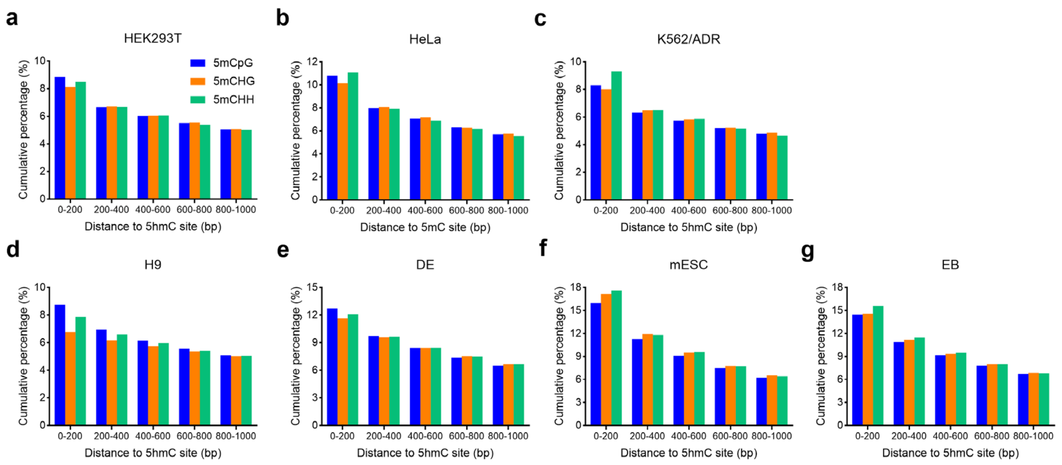

Figure S15. Cumulative distributions of distances from different types of 5mC sites (5mCpG, 5mCHG, and 5mCHH) to the nearest 5hmC sites, shown in 200 bp bins, across different cell lines. (a) HEK293T cells. (b) HeLa cells. (c) K562/ADR cells. (d) H9 cells. (e) DE cells. (f) mESCs. (g) EBs.

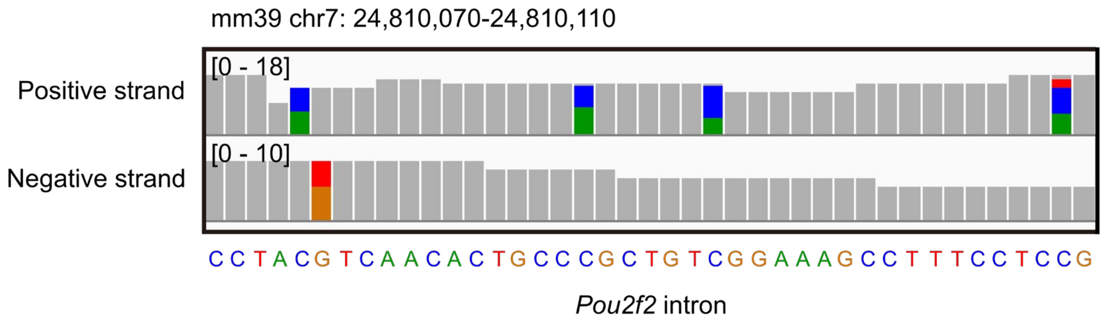

Figure S16. Representative single-base-resolution view of clustered 5mC sites in EB genome.

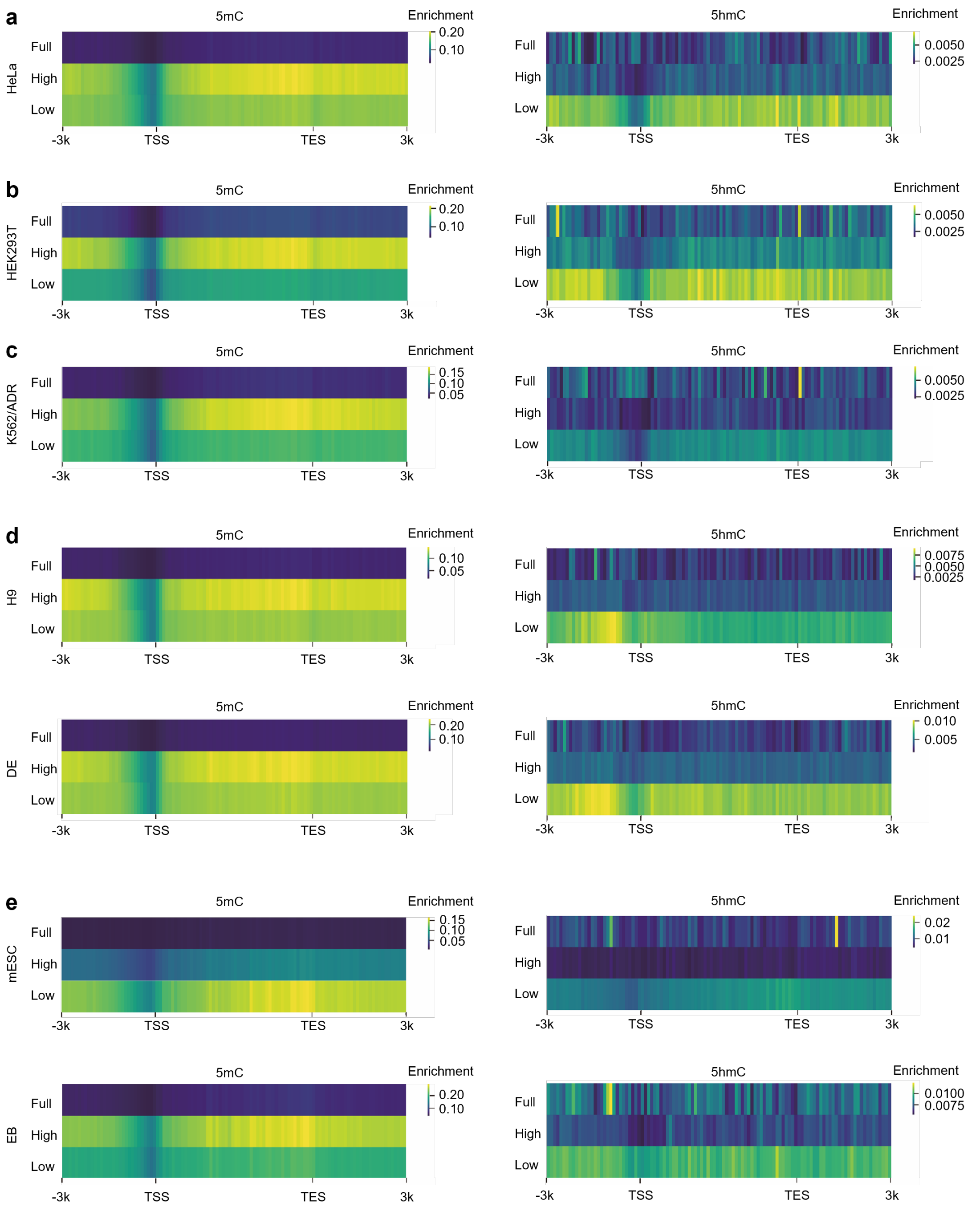

Figure S17. Heat maps revealing the distributional densities of 5mC and 5hmC with differential modification levels around TSS and TES in diverse cell lines. (a) HeLa cell. (b) HEK293T cell. (c) K562/ADR cell. (d) Comparison between H9 and DE cells. (e) Comparison between mESC and EB cells. Modification levels were classified into three groups: 10%–50% (low), 50%–90% (high), and over 90% (full).

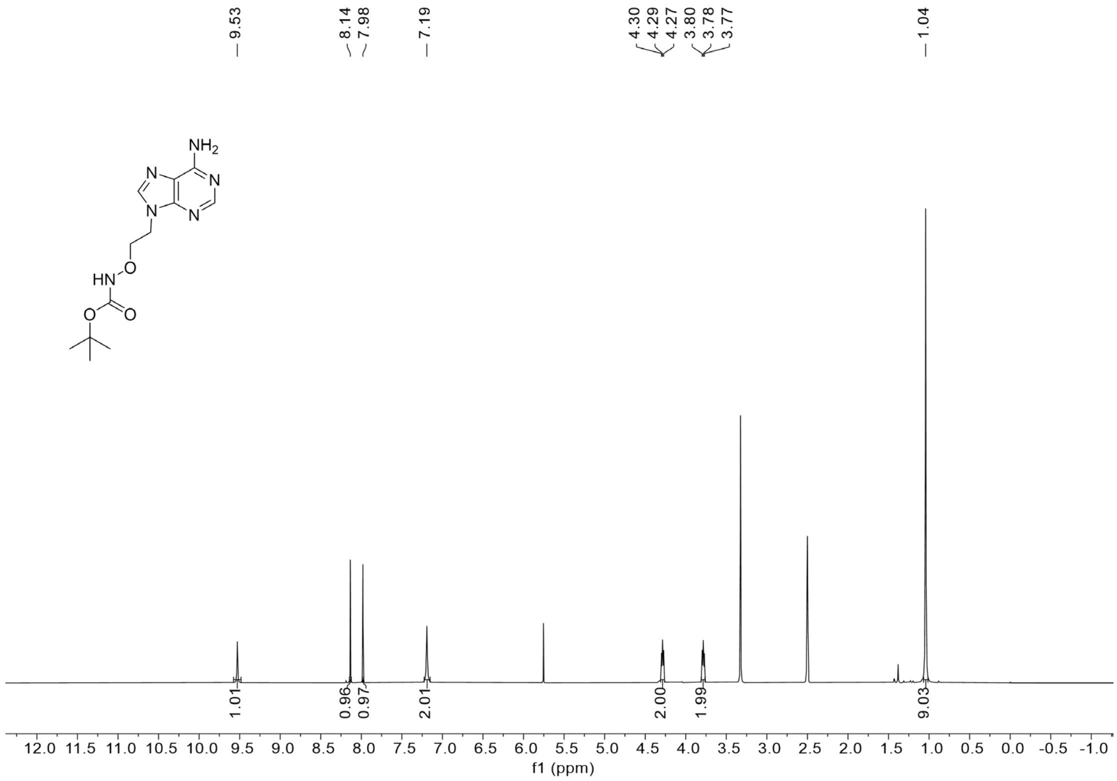

Figure S18. ^{1}H NMR (400 MHz) spectrum of A-Boc in DMSO-d_6.

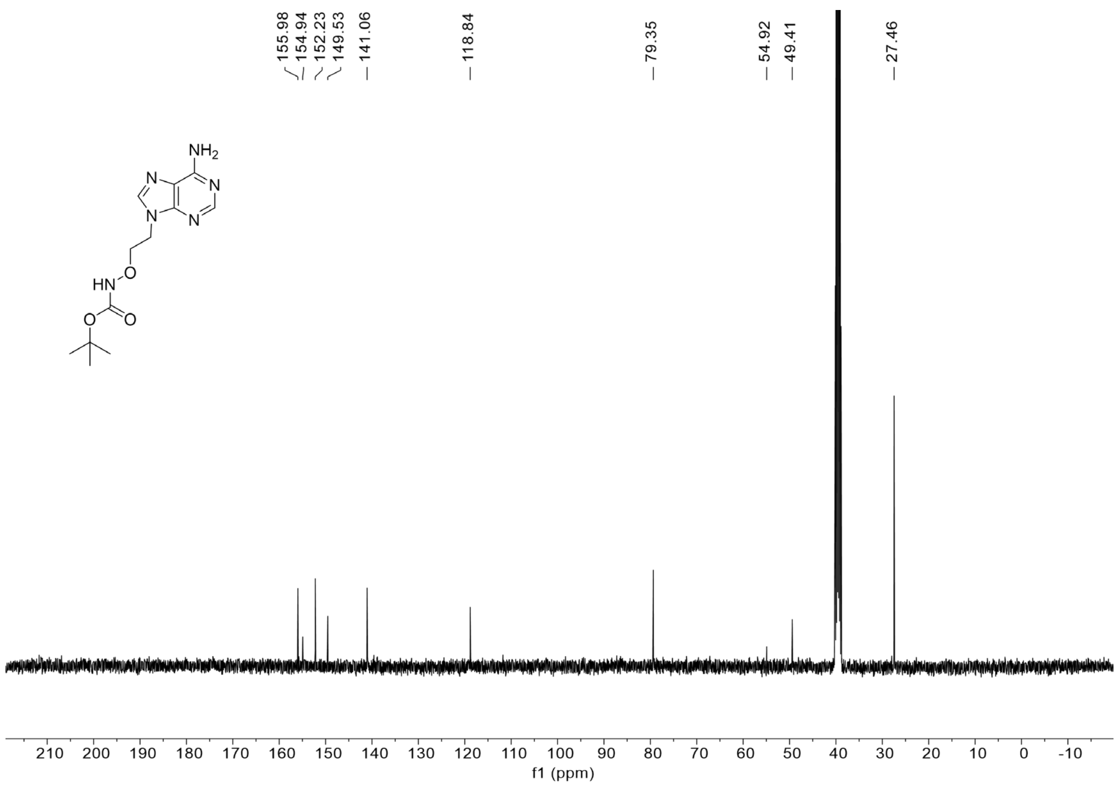

Figure S19. ^{13}C NMR (101 MHz) spectrum of A-Boc in DMSO-d_6.

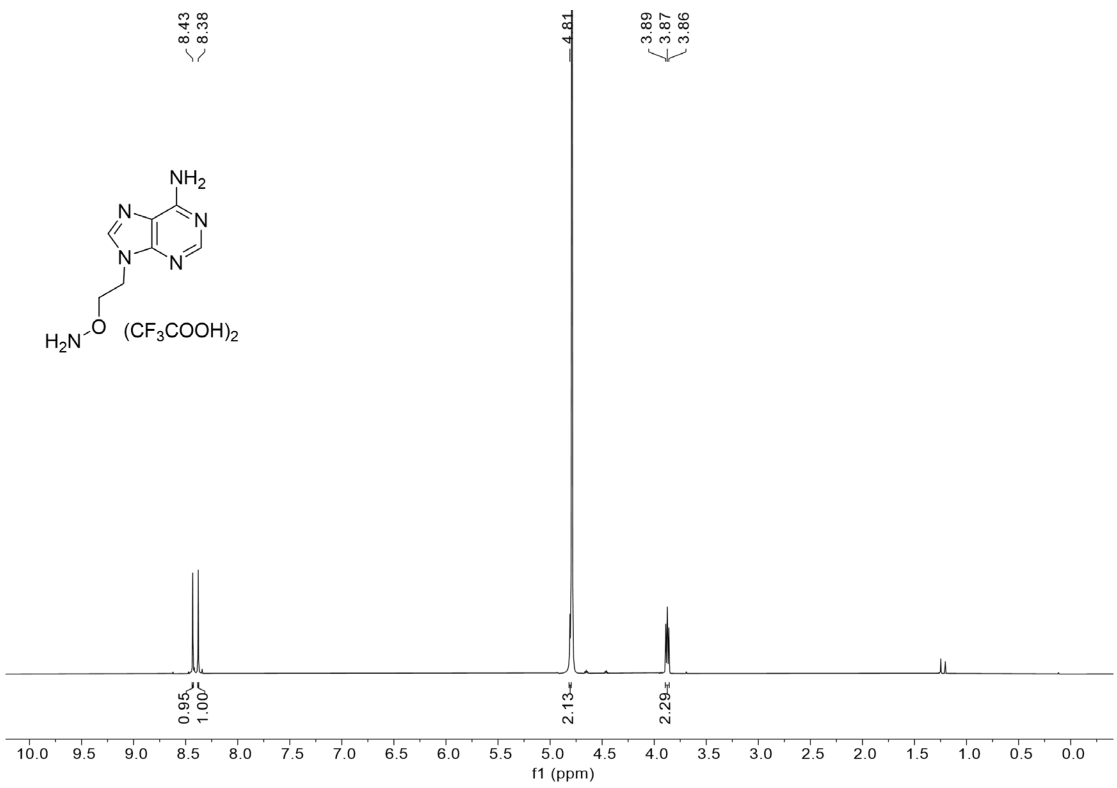

Figure S20. ^{1}H NMR (400 MHz) spectrum of A-Ha in D_2O.

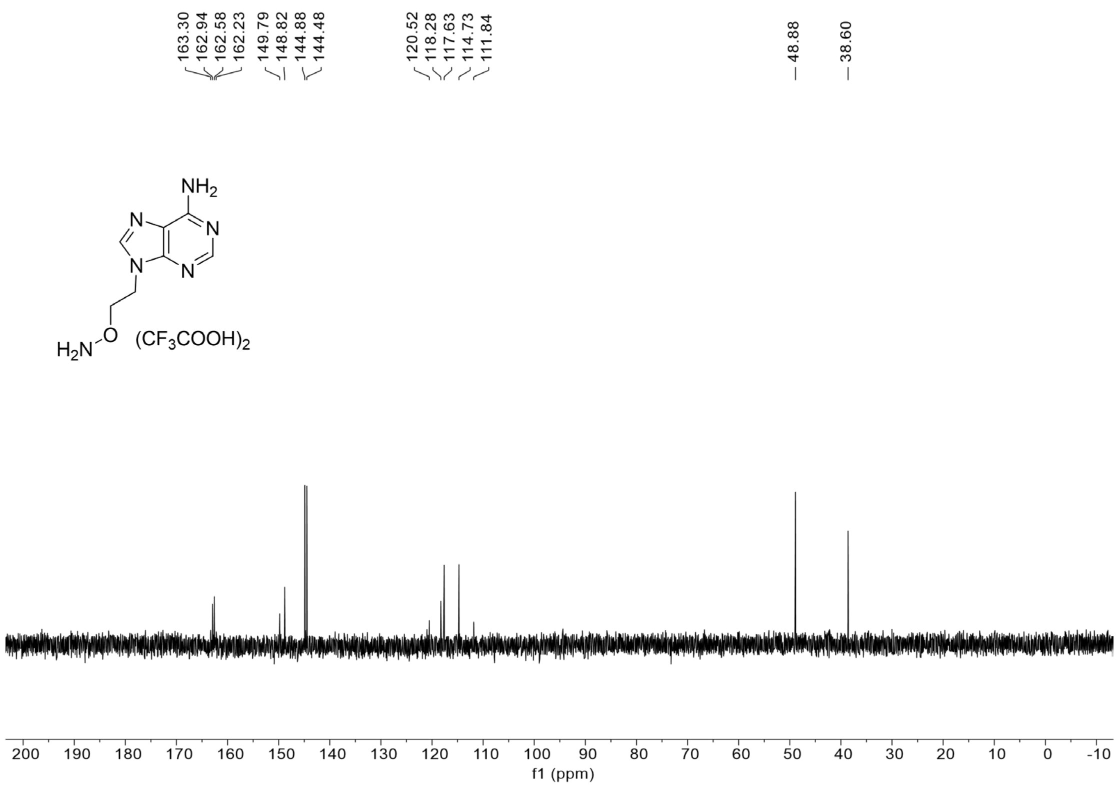

Figure S21. ^{13}C NMR (101 MHz) spectrum of A-Ha in D_2O.

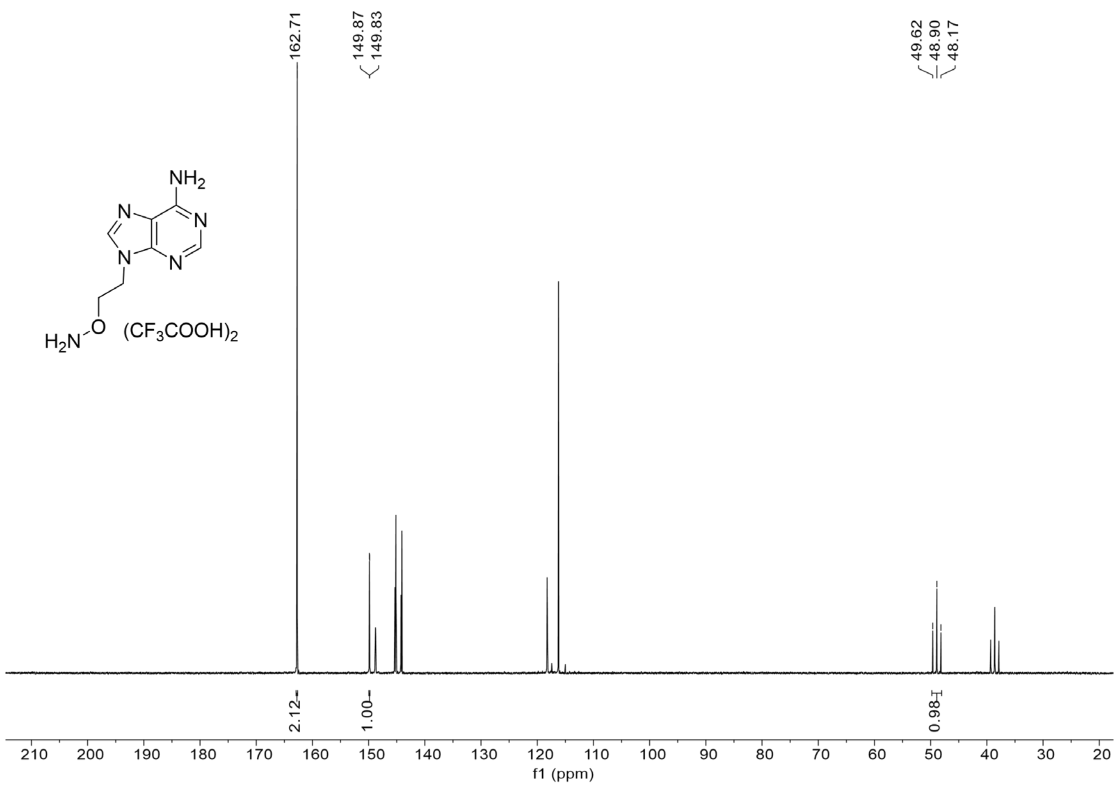

Figure S22. Quantitative ^{13}C NMR (800 MHz, fluorine decoupling) spectrum of A-Ha in D_2O.

Supplementary Tables

Table S1. Model sequences

Model DNA	Sequence (5'-3')
ODN1-dU	GACTCAAdUAGCCGTA
ODN2-dU	CGATCGAdUTCAGTCT
ODN3-dU	AGATCTdUCTGAGCAT
ODN4-dU	GTCACCAGdUGAGGTG
ODN5-dU	TGAdUCGACTCAGTCA
ODN6-dU	GTCACTCACTAGdUTA
ODN7-dU	CdUATGCATGAGCTAC
ODN8-1U	CCGGACTAGTCTGAATTATTGdUGTGTTCATGACCTAGGCG
ODN8-2U	CCGGACTAGTCdUGAATTATTGdUGTGTTCATGACCTAGGCG
ODN8-U2	CCGGACTAGTCTGAATTATdUGdUGTGTTCATGACCTAGGCG
ODN8-3U	CCGGACTAGTCdUGAATTATTGdUGTGTTCAdUGACCTAGGCG
ODN8-U3	CCGGACTAGTCTGAATTATdUGdUGdUGTTCATGACCTAGGCG
ODN8-U-R	CGCCTAGGTCAdUGAACACACAATAATTCAGACTAGTCCGG
NN-dU-NN-F	TCCTCCTACATCATTCCTCTCTAACCCCTTATATACGTTCGCCTTATCG GATCNNdUNNGTACTTAGAATCAATTGAGTGATTGAAGGTAGTTAGT GGTGGTAGA
NN-dU-NN-R	TCTACCACCACTAACTACCTTCAATCACTCAATTGATTCTAAGTACNN ANNGATCCGATAAGGCGAACGTATATAAGGGGTTAGAGAGGAATGA TGTAGGAGGA
ss-ODN9-caC-F	HEX- TTCTTCTACATCATCTCCCTCTAACCTCCTATATTA5caCATTATAATAA ATTAGGTAGATTAGAGAGTAGTAGTGGTGAGGA
ss-ODN9-R	TCCTCACCACTACTACTCTCTAATCTACCTAATTTATTATAATGTAATAT AGGAGGTTAGAGGGAGATGATGTAGAAGAA
ss-ODN9-F	TTCTTCTACATCATCTCCCTCTAACCTCCTATATTACATTATAATAAATT AGGTAGATTAGAGAGTAGTAGTGGTGAGGA
ss-ODN10-mC-F	GAA5mCGCTATGAGGACATGGCAGCCTTC
ss-ODN10-R	GAAGGCTGCCATGTCCTCATAGCGTTC

ss-ODN11-hmC-F	TCCTCCTACATCATTCCTCTCTAACCCCTTATATACGTTCGCCTTATCGGATC5hmCGTACTTAGAATCAATTGAGTGATTGAAGGTAGTTAGTGGTGGTAGA
ss-ODN11-C-F	TCCTCCTACATCATTCCTCTCTAACCCCTTATATACGTTCGCCTTATCGGATCCGTACTTAGAATCAATTGAGTGATTGAAGGTAGTTAGTGGTGGTAGA
ss-ODN11-R	TCTACCACCACTAACTACCTTCAATCACTCAATTGATTCTAAGTACGGATCCGATAAGGCGAACGTATATA
ss-ODN12-mC-F	CTAAATCTACTAAATCCTCTAAATCTATTCGGATT5mCGATCCGGAACGTGTAGGTAGTAGTAGTAGATATAAGATGATAGG
ss-ODN12-R	CCTATCATCTTATATCTACTACTACTACCTACACGTTCCGGATCGAATCCGAATAGATT
ss-ODN13-F-mChmC	CTAAATCTACTAAATCCTCTAAATCTATTCGGATT5hmCGATCCGGACTAGTCAAGCTTATCGGCTATTCAGGACCTAGG5mCGCACGTGTAGGTAGTAGTAGTAGATATAAGATGATAGG
ss-ODN13-R-C	CCTATCATCTTATATCTACTACTACTACCTACACGTGCGCCTAGGTCCTGAATAGCCGATAAGCTTGACTAGTCCGGATCGAATCCGAATAGATTTAGAGGATTTAGTAGATTTAG
ss-ODN13-F-mC	CTAAATCTACTAAATCCTCTAAATCTATTCGGATTCGATCCGGACTAGTCAAGCTTATCGGCTATTCAGGACCTAGG5mCGCACGTGTAGGTAGTAGTAGTAGATATAAGATGATAGG
ss-ODN13-F-hmC	CTAAATCTACTAAATCCTCTAAATCTATTCGGATTCGATCCGGACTAGTCAAGCTTATCGGCTATTCAGGACCTAGG5hmCGCACGTGTAGGTAGTAGTAGTAGATATAAGATGATAGG
ss-ODN13-F-C	CTAAATCTACTAAATCCTCTAAATCTATTCGGATTCGATCCGGACTAGTCAAGCTTATCGGCTATTCAGGACCTAGGCGCACGTGTAGGTAGTAGTAGTAGATATAAGATGATAGG

Table S2. Sequencing and mapping performance of BRIGHT-seq compared to other technologies in gDNA from mESC

Methods	Unique mapping reads	Clean reads	Unique mapping rate	Duplication rate	Input DNA amount	PCR Cycles
BRIGHT-seq 5mC rep1	625,122,702	470,887,041	82.92%	29.65%	250 ng	16
BRIGHT-seq 5mC rep2	844,844,610	600,654,065	82.99%	33.55%	250 ng	16

BRIGHT-seq 5hmC rep1	618,872,886	469,394,202	82.09%	29.45%	250 ng	16
BRIGHT-seq 5hmC rep2	836,800,882	598,909,502	82.20%	33.36%	250 ng	16
EM-seq rep1	227,407,748	157,315,098	66.90%	30.82%	100 ng	11
EM-seq rep2	266,877,736	175,363,287	69.70%	34.29%	100 ng	11
SIMPLE-seq 5mC [3]	782,164,031	184,163,140	80.82%	19.94%	Not provided	4-6
SIMPLE-seq 5hmC [3]	782,164,031	438,584,316	80.82%	27.67%	Not provided	4-6
TAPSβ [4]	662,301,088	589,228,992	81.67%	20.44%	100 ng	4
ACE-seq rep1	608,496,454	383,796,883	75.10%	36.93%	100 ng	10
ACE-seq rep2	424,028,440	308,962,470	74.40%	27.14%	100 ng	10

Table S3. Primer sequences

**Primer**	**Sequence (5'-3')**
Model-1-F	HEX-TTCTTCTACATCATCTCCCTCTAACCTCCT
Model-1-R	TCCTCACCACTACTACTCTCTAATCTACCT
Model-2-F	CTCTTTCCCTACACGACGCTCTTCCGATCTCCTCCTACATCATTCCTCTCTAACCCCT
Model-2-R	CTGGAGTTCAGACGTGTGCTCTTCCGATTCTACCACCACTAACTACCTTCAATCACTC
Model-3-F	CTCTTTCCCTACACGACGCTCTTCCGATCCTAAATCTACTAAATCCTCTAAATCTATTC
Model-3-R	CTGGAGTTCAGACGTGTGCTCTTCCGATCCCTATCATCTTATATCTACTACTACTACC
P7 primer	CAAGCAGAAGACGGCATACGAGAT

REFERENCES

1. Lu P, Yang J, Li M *et al*. A desert lncRNA *HIDEN* regulates human endoderm differentiation via interacting with IMP1 and stabilizing *FZD5* mRNA. *Genome Biol* 2023; **24**: 92.
2. Slyvka A, Mierzejewska K, Bochtler M. Nei-like 1 (NEIL1) excises 5-carboxylcytosine directly and stimulates TDG-mediated 5-formyl and 5-carboxylcytosine excision. *Sci Rep* 2017; **7**: 9001.
3. Bai D, Zhang X, Xiang H *et al*. Simultaneous single-cell analysis of 5mC and 5hmC with SIMPLE-seq. *Nat Biotechnol* 2025; **43**: 85–96.
4. Liu Y, Hu Z, Cheng J *et al*. Subtraction-free and bisulfite-free specific sequencing of 5-methylcytosine and its oxidized derivatives at base resolution. *Nat Commun* 2021; **12**: 618.
5. Zhang Y, Park C, Bennett C *et al*. Rapid and accurate alignment of nucleotide conversion sequencing reads with HISAT-3N. *Genome Res* 2021; **31**: 1290–5.
6. Picardi E and Pesole G. REDItools: high-throughput RNA editing detection made easy. *Bioinformatics* 2013; **29**: 1813–4.
7. Krueger F and Andrews SR. Bismark: a flexible aligner and methylation caller for Bisulfite-Seq applications. *Bioinformatics* 2011; **27**: 1571–2.
8. Langmead B and Salzberg SL. Fast gapped-read alignment with Bowtie 2. *Nat Methods* 2012; **9**: 357–9.
9. Thorvaldsdóttir H, Robinson JT, Mesirov JP. Integrative Genomics Viewer (IGV): high-performance genomics data visualization and exploration. *Brief Bioinform* 2013; **14**: 178–92.
10. ENCODE Project Consortium. An integrated encyclopedia of DNA elements in the human genome. *Nature* 2012; **489**: 57–74.
11. Zhang J, Lee D, Dhiman V *et al*. An integrative ENCODE resource for cancer

genomics. *Nat Commun* 2020; **11**: 3696.

12. Lee D, Zhang J, Liu, J *et al*. Epigenome-based splicing prediction using a recurrent neural network. *Plos Comput Biol* 2020; **16**: e1008006.
13. Ramírezv F, Ryan DP, Grüning B *et al*. deepTools2: a next generation web server for deep-sequencing data analysis. *Nucleic Acids Res* 2016; **44**: W160–5.
14. Gao T and Qian J. EnhancerAtlas 2.0: an updated resource with enhancer annotation in 586 tissue/cell types across nine species. *Nucleic Acids Res* 2020; **48**: D58–64.
15. Akalin A, Kormaksson M, Li S *et al*. methylKit: a comprehensive R package for the analysis of genome-wide DNA methylation profiles. *Genome Biol* 2012; **13**: R87.
16. Heinz S, Benner C, Spann N *et al*. Simple combinations of lineage-determining transcription factors prime *cis*-regulatory elements required for macrophage and B cell identities. *Mol Cell* 2010; **38**: 576–89.
17. Sherman BT, Hao M, Qiu J *et al*. DAVID: a web server for functional enrichment analysis and functional annotation of gene lists (2021 update). *Nucleic Acids Res* 2022; **50**: W216–21.
